# New Plant Growth-Promoting, Chromium-Detoxifying *Microbacterium* Species Isolated From a Tannery Wastewater: Performance and Genomic Insights

**DOI:** 10.3389/fbioe.2020.00521

**Published:** 2020-07-03

**Authors:** Rania Ouertani, Awatef Ouertani, Mouna Mahjoubi, Yosra Bousselmi, Afef Najjari, Hanene Cherif, Asma Chamkhi, Amor Mosbah, Hechmi Khdhira, Haitham Sghaier, Habib Chouchane, Ameur Cherif, Mohamed Neifar

**Affiliations:** ^1^Univ. Manouba, ISBST, BVBGR-LR11ES31, Biotechpole Sidi Thabet, Sidi Thabet, Tunisia; ^2^Laboratory of Microorganisms and Active Biomolecules, MBA-LR03ES03, Faculty of Sciences of Tunis, University of Tunis El Manar, Tunis, Tunisia; ^3^Management Environment Responsible in Tanneries Mégisseries du Maghreb, TMM, Grombalia, Tunisia; ^4^Laboratory “Energy and Matter for Development of Nuclear Sciences” (LR16CNSTN02), National Center for Nuclear Sciences and Technology (CNSTN), Sidi Thabet Technopark, Sidi Thabet, Tunisia

**Keywords:** actinobacterium, bioremediation, genome annotation, heavy metals, plant growth promotion, tannery wastewater

## Abstract

Hexavalent chromium [Cr(VI)], widely generated by tannery activities, is considered among the most toxic substances and causes a serious damage for the environment and for human health. Interestingly, some microorganisms have a potential of bioremediation of chromium-contaminated wastewaters and soils through the reduction of Cr(VI) (soluble and harmful form) into Cr(III) (stable and non-toxic form). Here, we present the full genome sequence of a novel heavy-metal-resistant, plant growth-promoting bacterium (PGPB), *Microbacterium metallidurans* TL13, which was isolated from a Tunisian leather industry. The strain TL13 was resistant to many heavy metals, such as chromium, copper, nickel, cobalt, and arsenic. The 50% TL13 growth inhibitory concentration (IC_50_) values of HgCl_2_, CoCl_2_, K_2_Cr_2_O_7_, CuSO_4_, NiCl_2_, FeSO_4_, and Na_2_HAsO_4_ are 368, 445, 676, 1,590, 1,680, 4,403, and 7,007 mg/L, respectively, with the following toxicity order: HgCl_2_ > CoCl_2_ > K_2_Cr_2_O_7_ > CuSO_4_ > NiCl_2_ > FeSO_4_ > Na_2_HAsO_4_. This new strain was also able to promote the growth of the hybrid tomato (Elika F1) under chromium metal stress. Its whole genome sequence length was estimated to be 3,587,460 bp (3,393 coding sequences) with a G + C content of 70.7%. Functional annotation of the genome of TL13 revealed the presence of open reading frames (ORFs) involved in adaptation to metal stress, such as the chromate transport protein, cobalt–zinc–cadmium resistance protein, copper resistance protein, copper responsive transcriptional regulator, multidrug resistance transporters, arsenical resistance operon repressor, arsenate reductase, arsenic resistance protein, mercuric resistance operon regulatory protein, mercuric ion reductase, and organomercurial lyase. Moreover, genes for the production of glutathione peroxidase, catalase, superoxide dismutase, and thioredoxin reductase, which confer a higher tolerance to oxidative/metal stresses, were identified in TL13 genome. In addition, genes for heat shock tolerance, cold shock tolerance, glycine-betaine production, mineral phosphate solubilization, ammonia assimilation, siderophores, exopolysaccharides, polyketides, and lytic enzymes (cellulase, chitinase, and proteases) production that enable bacteria to survive biotic/abiotic stress and to promote plant growth and health were also revealed. Based on genome analysis and experimental approaches, strain TL13 appears to have evolved from various metabolic strategies and could play a role in ensuring sustainable environmental and agricultural systems.

## Introduction

The leather industry plays an important role in the world's economy. This industry uses a raw material that is originally a livestock coproduct. Leather making consists of converting putrescible hides and skins into a more resistant commercial leather (Fang et al., 2017). In fact, this process comprises three main operations: (i) beamhouse operation, which consists of several steps aimed to eliminate all components other than collagen; (ii) tanning operation, which provides resistant leather using chromium; and (iii) finishing operation, which consists of giving the treated leather its last aspect ready for commercialization. During the process of leather making, several baths containing chemicals harmful to the environment are used in order to treat hides and skins. Indeed, spilled water has a significant pollutant load (Thanikaivelan et al., 2004). Around 30 m^3^ of effluents is generated for every ton of hides and skins, which have significant impacts on environments and human health due to the presence of heavy metals especially chromium (Suthanthararajan et al., 2004). The toxic form of this metal [Cr(VI)] seems to be the cause of the contamination of aquatic and terrestrial ecosystems and also the cause of several diseases such as cancer, particularly lung cancer (Pradhan et al., 2016).

Despite its toxicity (Balmer, 2018), hexavalent chromium [Cr(VI)] could be removed with an ecofriendly method using microorganisms, a process called bioremediation. Many studies have proven the effectiveness of bacteria and fungi for the reduction of Cr(VI) in chromium-contaminated effluents and soils and the ability to transform it into Cr(III) (Chai et al., 2019). Several bacterial strains are known as potential chromium reducers, such as *Enterobacter* sp. HU38, *Pantoea stewartii* ASI11 (Ashraf et al., 2018), *Cellulosi microbium* sp. (Bharagava and Mishra, 2018), *Brucella* sp. K12 (Maqbool et al., 2015), *Escherichia coli* (Learman et al., 2011), *Amphibacillus* sp. KSUCr3 (Ibrahim et al., 2012), *Pseudomonas aeruginosa* CCTCC AB93066 (Kang et al., 2014), and *Bacillus firmus* KUCr1 (Sau et al., 2010). *Microbacterium* strains, being able to tolerate or resist various heavy metals such as chromium, arsenic, nickel, and cadmium, have been found to be suitable bacteria for bioremediation (Soni et al., 2014; Gutiérrez et al., 2015; Wu et al., 2015; Lun et al., 2016; Elahi et al., 2019; Kumar and Saini, 2019). *Microbacterium* species have been recently described as efficient plant growth-promoting bacterium (PGPB) although under biotic and abiotic stresses (Manzanera et al., 2015; Jana et al., 2017; Passari et al., 2019a).

In recent years, several bacterial whole genomes were sequenced in order to identify loci involved in metal resistance, particularly chromium reduction and resistance. Genomic annotations indicated that *chrR* and *yieF* are implicated in the mechanism of chromate reduction. In fact, some bacterial strains, like *E. coli*, use YieF reductase to reduce Cr(VI) into Cr(III). Other species, like *Pseudomonas putida*, are known for their chromium reduction through the ChrR reductase (Ramirez-Diaz et al., 2008; Learman et al., 2011; Ahemad and Kibret, 2014; Henson et al., 2015; Baldiris et al., 2018). Otherwise, *M. laevaniformans* strain OR221 was able to resist some heavy metals, but the annotation of its genome after sequencing suggested the absence of both ChrR and YieF (Brown et al., 2012). In this paper, we describe the identification of a novel heavy-metal-resistant, PGPB *M. metallidurans* TL13, isolated from a tannery effluent. TL13 could prove to be a suitable candidate for the bioremediation of heavy-metal-contaminated soils and wastewaters and also could be used as a biofertilizer due to its traits. The complete genome sequencing and annotation of TL13 were conducted, and experimental data were collected, in order to better understand its mechanisms of chromium resistance and reduction and genes involved in stress resistance and PGP activities.

## Materials and Methods

### Reagents and Chemicals

All chemicals and reagents used in this study are of pure analytical grade and available commercially.

### Isolation and Characterization of a Novel Heavy-Metal-Resistant Bacterium

In this study, a heavy-metal-resistant bacterium strain TL13 was isolated from tannery (TMM) wastewater using tryptic soy agar (TSA). Water content, total organic carbon (TOC), organic content, and total heavy metal content from the sludge generated in TMM wastewater treatment plants were determined by gravimetry, calcination, colorimetry, and inductively coupled plasma atomic emission spectroscopy (ICP-OES) according to the standards ISO 12880: 2000, Rodier, NF ISO 14235: 1998 and ISO 1188: 2007, respectively (Zeng et al., 2016).

Analysis of bacterial resistance to heavy metals was carried out on TSA medium supplemented with increasing concentrations (50–2,500 mg/L) of iron sulfate (FeSO_4_), copper sulfate (CuSO_4_), cobalt chloride (CoCl_2_), disodium arsenate (Na_2_ HAsO_4_), potassium dichromate (K_2_Cr_2_O_7_), mercury chloride (HgCl_2_), and nickel chloride (NiCl_2_). The spot inoculation method was used to involve in triplicate 10 μl of bacterial culture suspension (10^8^ CFU/mL) (Fu et al., 2016). Bacterial cultures were incubated for 24 h at 30°C to determine minimum inhibitory concentrations (MIC) (Davis, 2005). A positive control was used by inoculating bacterial suspension on agar plates without metal. The half maximal inhibitory concentration (IC_50_) was determined using experiments conducted in tryptic soy broth (TSB) medium at 30°C with shaking at 120 rpm (Fassy et al., 2017). Bacterial growth was measured after 24 h on a UV–visible (UV-Vis) spectrophotometer at 600 nm. All experiments (solid and liquid) were performed under alkaline conditions (pH 9). Metal reduction was determined by inductively coupled plasma mass spectrometry. Bacterial cell free extracts were recovered after a 10 min of centrifugation at 10,000 rpm and then filtered using 0.22 μm Whatman filter paper.

### Plant Growth-Promoting Activities

#### *In vitro* Screening of TL13 for Its Plant Growth-Promoting Activities

The screening of TL13 strain for various *in vitro* plant growth-promoting activities was performed by adopting standard methods (Hassen et al., 2018). Inorganic phosphate-solubilizing activity and growth on nitrogen free medium was estimated by the method of Pikovskaya (1948) and Jensen (1942), respectively. To detect the ability of TL13 strain to produce siderophore, chrome azurol sulfonate (CAS) agar solid medium was used (Alexander and Zuberer, 1991). Cellulase activity was tested on minimal medium amended with 1% carboxymethyl cellulose (CMC) (Souii et al., 2018). The protease production was screened by skim milk agar (Ouertani et al., 2018). The ability of TL13 to produce indole-3-acetic acid (IAA) and exopolysaccharides (EPS) was assessed as indicated by Penrose and Glick (2003) and Naseem et al. (2018), respectively.

#### *In vivo* Plant Growth Promotion Assays

*In vivo* plant growth promotion and tomato seed germination assays were performed as described by Passari et al. (2019a) with slight modification. Elika F1 tomato seeds were surface sterilized with 1% sodium hypochlorite solution for 20 min and subsequently rinsed three times with sterile distilled water. Disinfected Elika F1 tomato seeds were immersed for 2 h in a TL13 bacterial suspension (10^8^ CFU/mL). Seeds immersed in sterile distilled water served as a control. A sterile Whatman paper was soaked in a chromium solution at different concentrations (0, 100, and 500 mg/L) and deposited in Petri dishes. Seeds were then gently deposited on Whatman filter paper under appropriate conditions, incubated in darkness at 30°C for 8 days and irrigated every day with sterile distilled water. Germination percentages were calculated by dividing total germinated seeds to total number of seeds that were planted (Passari et al., 2019a,b). The experiment was repeated three times using 20 Elika F1 tomato seeds per germination condition. Different parameters, including shoot length, shoot fresh, and dry weights, were measured.

#### Antibiotic Sensitivity Test

The Kirby–Bauer test, known as the disk-diffusion method, was performed to determine antibiotic sensitivity of TL13 strain (Bauer et al., 1966). A pure culture of TL13 strain was diluted using saline solution and adjusted to 10^8^ CFU/mL. Bacterial inoculum was then streaked on Mueller Hinton agar using a sterile swab. Antibiotics used were cefoxitin (FOX, 30 μg), pristinamycin (PTN, 15 μg), neomycin (NE, 30 μg), rifampicin (RD, 30 μg), norfloxacin (NOR, 5 μg), streptomycin (S, 10 μg), erythromycin (E, 15 μg), cefotaxime (CTX, 30 μg), tetracycline (TET, 30 μg), vancomycin (VAN, 30 μg), ceftazidime (CAZ, 30 μg), spiramycin (SP, 100 μg), and oxytetracyclin (OT, 30 μg). Antibiotic impregnated disks were placed on agar plates and incubated for 72 h at 30°C. Bacterial resistance or sensitivity was determined by measuring the zone of inhibition.

### Genome Sequencing, Assembly, and Annotation of TL13 Strain

Genomic DNA extraction of TL13 strain was performed by sodium dodecyl sulfate–proteinase K treatment (Neifar et al., 2019). Purified genomic DNA was sequenced on an Illumina MiSeq platform (MRDNA, USA) yielding 2 ×250 bp paired-end reads. The Prinseq-lite software was used for trimming the 4,320,030 paired reads by discarding low-quality sequences with a score lower than 23 (Mahjoubi et al., 2019). Genome assembly was then performed using the SPAdes algorithm (Bankevich et al., 2012). Genome annotation was done using the Integrated Microbial Genomes/Expert Review (IMG/ER) database (Markowitz et al., 2009) and the Rapid Annotations using Subsystems Technology (RAST) server (Aziz et al., 2008). Annotated protein sequences were downloaded from the National Center for Biotechnology Information (NCBI) (Galperin et al., 2015) and the Kyoto Encyclopedia of Genes and Genomes (KEGG) (Ogata et al., 1999) databases.

### Phylogenetic Analysis

The 16S ribosomal DNA (rDNA) gene sequence was annotated within the genome and compared to sequences of bacterial strains from the NCBI and EzBioCloud databases (Kim et al., 2012). The phylogenetic tree was inferred by the neighbor-joining method using the Molecular Evolutionary Genetics Analysis (MEGA 6) software (Kumar et al., 2016). A 95–96% average nucleotide identity (ANI) value was used as a genomic measure for prokaryotic species delineation (Richter et al., 2016) and was measured using the JSpecies Web Server (JSpeciesWS) available at http://jspecies.ribohost.com/jspeciesws. A circular genome map was drawn used CGView software (Grant and Stothard, 2008).

### Structural Analysis

The structures of enzymes involved in chromate reduction were predicted by the online structure prediction tool, Iterative Threading ASSEmbly Refinement server (I-TASSER) (Yang and Zhang, 2015; Ouertani et al., 2018). The confidence score (C score) was used to identify the best generated model, and MolProbity was applied to evaluate the refined model coordinates (Davis et al., 2007). Enzyme models were assessed using COACH software (Yang et al., 2013). A final analysis with CCP4mg molecular-graphics software was performed to validate the quality of the structures (McNicholas et al., 2011).

### Statistical Analysis

Experimental results were expressed as the mean value of three independent replicates ± the standard deviation (SD). Data were analyzed using one-way ANOVA with *post-hoc* Duncan's tests (SPSS 16.0 for Windows, SPSS Inc., USA). Statistical significance was determined as *p* <0.05.

### Nucleotide Sequence Accession Number

The Whole Genome Shotgun project was deposited at DDBJ/ENA/GenBank under the accession SZZQ00000000. The version reported in this work is SZZQ01000000 (https://www.ncbi.nlm.nih.gov/nuccore/SZZQ00000000).

## Results

### Characterization of a Heavy-Metal-Resistant, Plant Growth-Promoting Actinobacterium Isolated From a Tannery Wastewater

**Figure 1:**
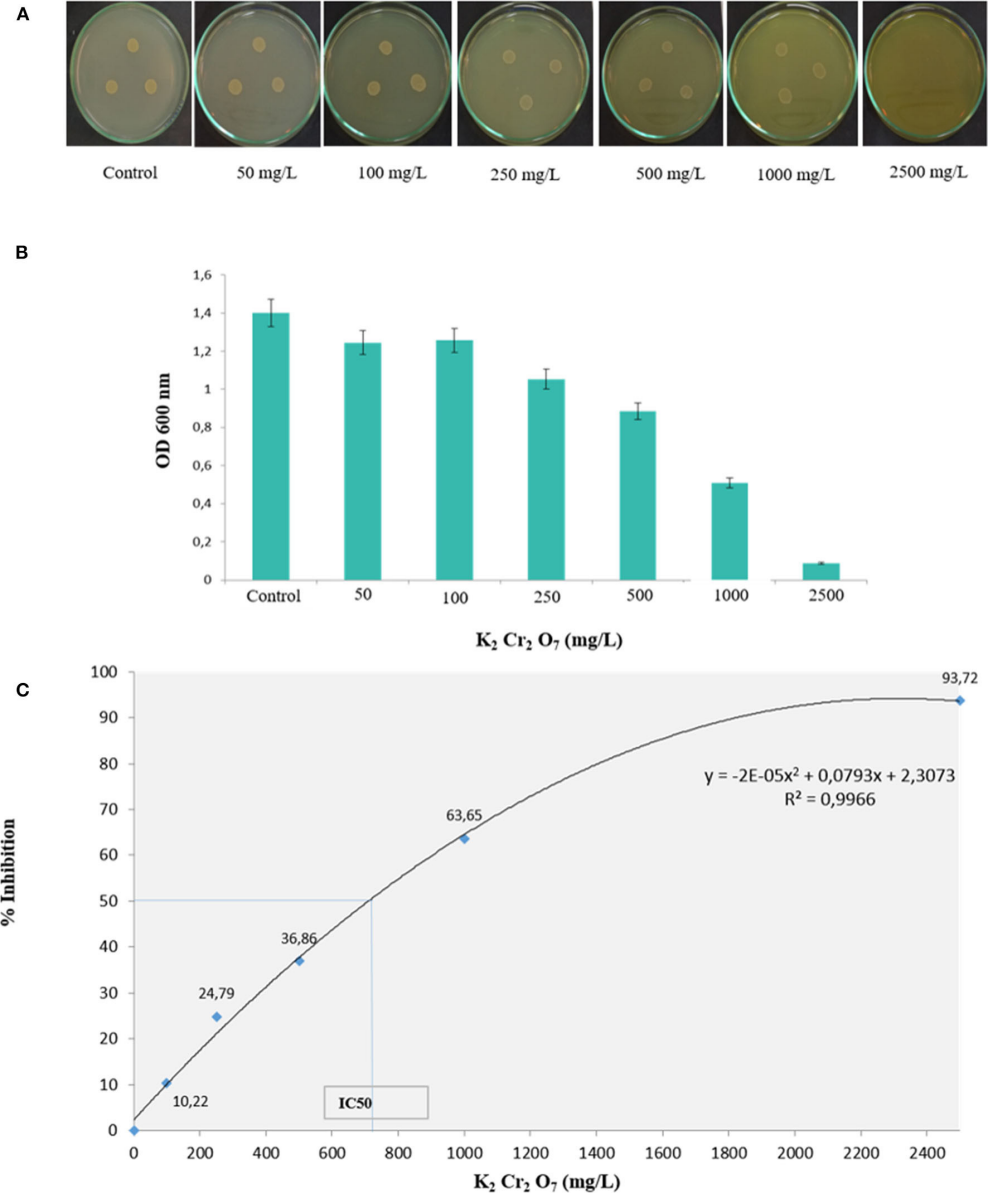
Effect of chromium concentration on TL13 bacterial growth on **(A)** tryptic soy agar (TSA) medium and **(B)** tryptic soy broth (TSB) medium; **(C)** chromium dose–response curve and determination of the IC_50_.

The physicochemical composition of the sludge collected from TMM industry is shown in Table 1. The content of chromium in sludge exceeds the thresholds for agriculture, according to national and international standards (Jakov, 2005). Qualitative analyses indicated that TL13 strain has the capacity to grow in the presence of K_2_Cr_2_O_7_ concentrations that could exceed 1,000 mg/L (Figure 1A). The bacterial growth was followed in the presence of K_2_Cr_2_O_7_ with variable concentrations ranging from 50 to 2,500 mg/L on TSB medium. As shown in Figure 1B, TL13 strain tolerated more than 1,000 mg/L of K_2_Cr_2_O_7_. The TL13 IC_50_ of K_2_Cr_2_O_7_ is equal to 676 mg/L (Figure 1C). ICP-MS analysis revealed that TL13 strain was able to reduce 71.68% of chromium [Cr(VI)] with initial concentration of 500 mg/L K_2_Cr_2_O_7_. Heavy metal resistance experiments indicated also that TL13 strain has the capacity to grow under high concentrations of FeSO_4_ and Na_2_HAsO_4_ (up to 2,500 mg/L), NiCl_2_ and CuSO_4_ (up to 1,000 mg/L), and CoCl_2_ and HgCl_2_ (up to 500 mg/L). IC_50_ values of HgCl_2_, CoCl_2_, K_2_Cr_2_O_7_, CuSO_4_, NiCl_2_, FeSO_4_, and Na_2_HAsO_4_ are 368, 445, 676, 1,590, 1,680, 4,403, and 7,007 mg/L, respectively (Table 2).

**Table 1:** Physicochemical characteristics of a TMM raw sludge sample.

**Parameters**	**Values**
Moisture (%)	68.2
Organic content (g/kg-dry sludge)	601
TOC (mg/kg-dry sludge)	353
Cadmium (mg/kg-dry sludge)	<0.6
Cobalt (mg/kg-dry sludge)	<5.0
Copper (mg/kg-dry sludge)	7.11
Iron (mg/kg-dry sludge)	16.4 ×10^3^
Lead (mg/kg-dry sludge)	7.52
Nickel (mg/kg-dry sludge)	47.05
Zinc (mg/kg-dry sludge)	561.7
Chromium (mg/kg-dry sludge)	26.2 ×10^3^
Arsenic (mg/kg-dry sludge)	<1.0
Selenium (mg/kg-dry sludge)	4.6
Antimony (mg/kg-dry sludge)	302.7
Tin (mg/kg-dry sludge)	<1.0
Silver (mg/kg-dry sludge)	12.86
Boron (mg/kg-dry sludge)	<1.0
Baryum (mg/kg-dry sludge)	33.04
Beryllium (mg/kg-dry sludge)	<1.0
Molybdenum (mg/kg-dry sludge)	<1.0
Titanium (mg/kg-dry sludge)	17.47
Mercury (mg/kg-dry sludge)	<1.0

**Table 2:** Heavy metals resistance of TL13 strain.

**Heavy metal resistance**		
**Metal**	**MIC ± SD (mg/L)**	**IC50 ± SD (mg/L)**
Chromium (K_2_Cr_2_O_7_)	1,000 ± 47	676 ± 33
Copper (CuSO_4_)	1,000 ± 42	1,590 ± 69
Nickel (NiCl_2_)	1,000 ± 50	1,680 ± 74
Cobalt (CoCl_2_)	500 ± 23	445 ± 19
Iron (FeSO_4_)	2,500 ± 113	4,403 ± 187
Mercury (HgCl_2_)	500 ± 21	368 ± 14
Arsenic (Na_2_HAsO_4_)	2,500 ± 107	7,007 ± 282

*Data are displayed as mean SD of three independent experiments performed in triplicate*.

**Figure 2:**
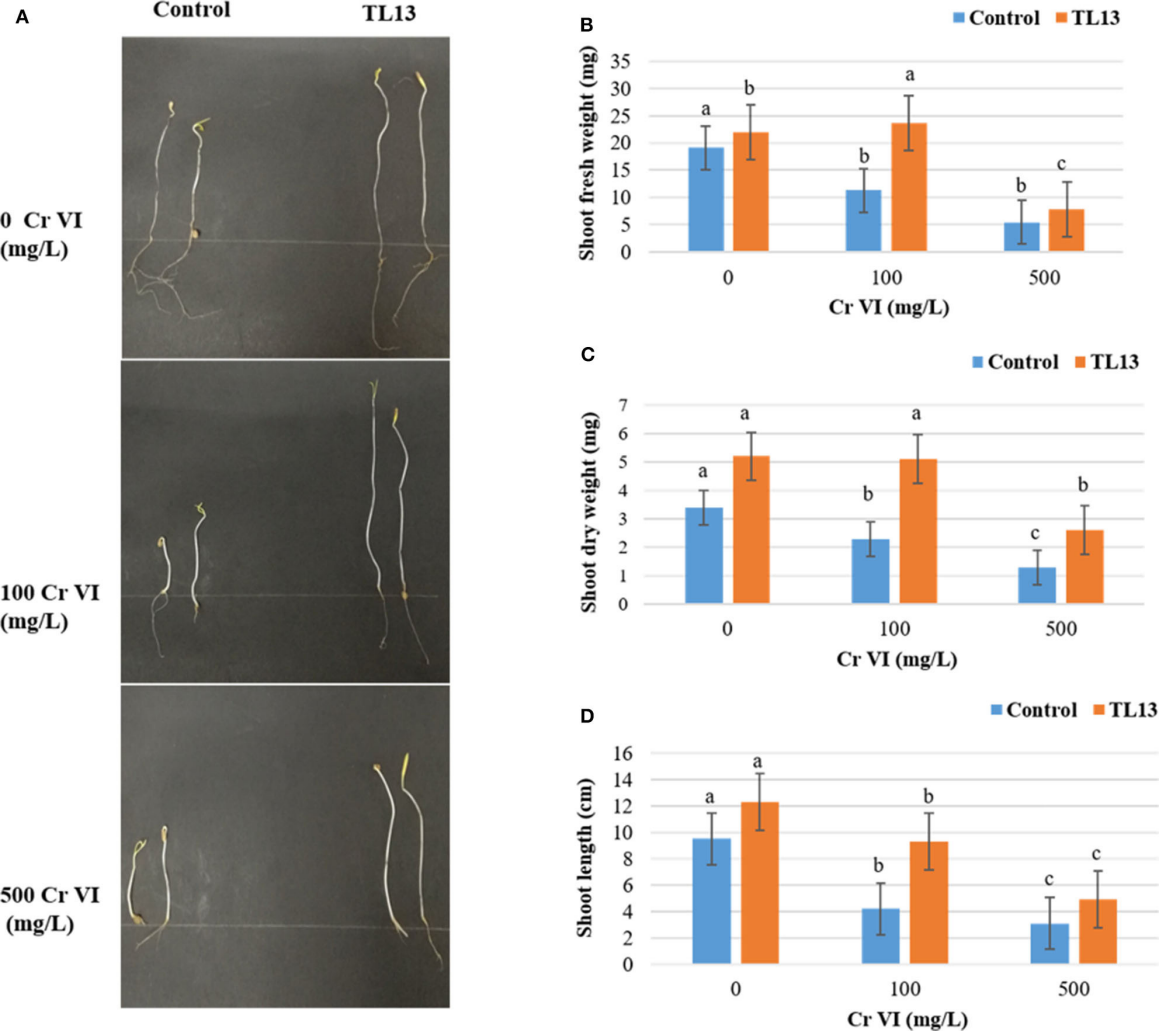
Effect of TL13 inoculation on the germination of tomato seeds under 0, 100, and 500 mg/L of Cr(VI). **(A)** View of the plantlets after 8 days of germination; **(B)** shoot fresh weight; **(C)** shoot dry weight; **(D)** shoot length. Values are means of (*n* = 3 per treatment); for each treatment, 20 tomato seeds were considered; error bars represent standard deviation. For each parameter, panels with different letters indicate significant differences at *P* <0.05 (ANOVA, Duncan *post-hoc* test).

The *in vitro* study of plant growth-promoting activities showed that TL13 strain was able to fix atmospheric nitrogen and to solubilize phosphate. The strain TL13 has also the capacity to produce siderophores, EPS, and cellulase as materialized by the presence of transparent halo around the colony in the corresponding culture media. Furthermore, the development of a pink color following the addition of Salkowski reagent to the culture supernatant showed the capacity of TL13 to produce IAA (Figure S1). The results of antibiograms (Figure S2, Table S1) showed the resistance of TL13 strain to cefotaxime, ceftazidime (antibiotics of β-lactam group), and norfloxacin (antibiotic of fluoroquinolone group). *M. metallidurans* TL13 was sensitive against all other tested antibiotics. Tomato *in vivo* experiments revealed positive effect of the TL13 strain on seeds germination under chromium stress. In fact, with simple visual inspection, the toxic effect of high levels of chromium and the promoting effect of TL13 on seeds germination were well distinguishable (Figure 2A). Tolerance to higher chromium levels was observed with TL13-treated seeds compared to control seeds since a significant increase in shoot fresh and dry weights and shoot length were recorded (Figures 2B–D).

### Phylogenetic Analysis and Genomic Insights Inferred From TL13 Genome Sequence

**Figure 3:**
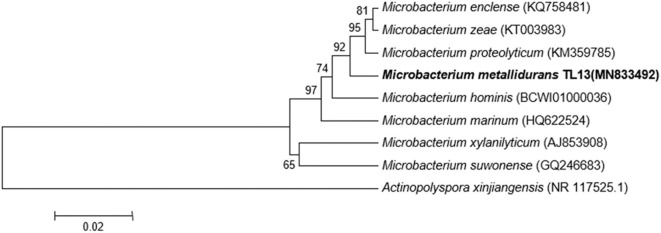
Phylogenetic tree highlighting the position of *M. metallidurans* TL13 among related taxa within the genus *Microbacterium* based on 16S rDNA gene sequences. Evolutionary distances were calculated using the method of maximum composite likelihood, and the topology was inferred using the neighbor-joining method using MEGA 7. Numbers on the nodes present % bootstrap values based on 500 replicates. Scale bar represents 0.02 substitutions per site. The 16S rDNA gene sequence of *Actinopolyspora xinjiangensis* was arbitrarily chosen as the outgroup to define the root of the tree.

**Figure 4:**
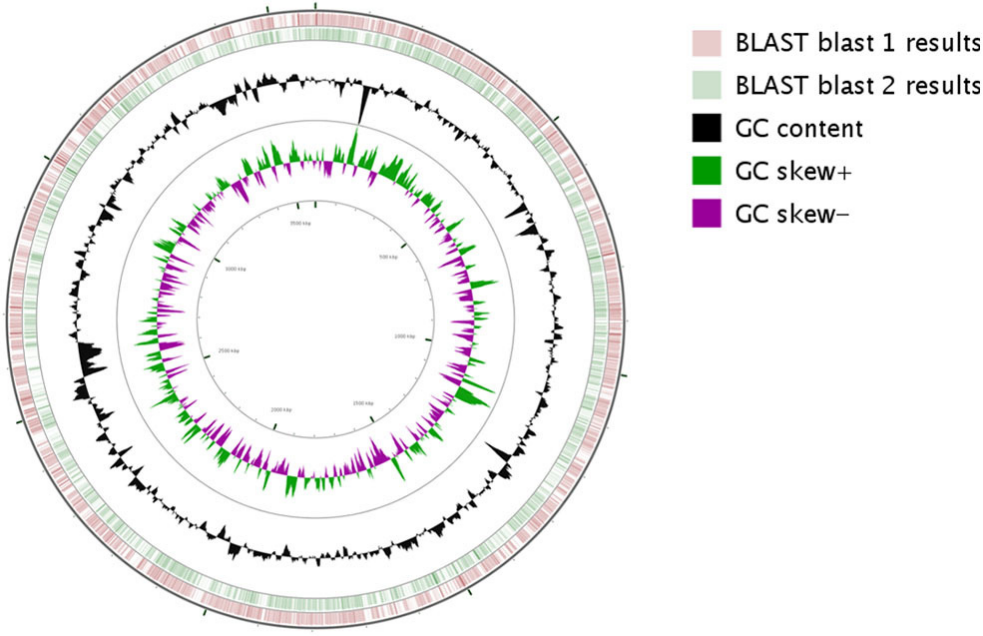
Circular representation of *M. metallidurans* TL13 genome generated by CG viewer. The innermost rings depict GC content (Black) and GC Skew (purple/green) followed by concentric rings of query sequences colored according to BLAST identity. The outermost rings depict genomes of the following microbes *M. enclense* NIO-1002 (pink) and *M. hominis* TPW29 (green).

Based on the proposed rule of using 16S rDNA sequence similarity threshold value of 98.65% for species delineation (Kim et al., 2014), TL13 and *Microbacterium proteolyticum* (RZ36) should belong to the same species as their 16S rDNA sequences exhibited an identity of 99.3%. However, this taxonomic result was not corroborated by the phylogenetic tree (Figure 3) as both strains are not sister taxa and do not form a distinct clade for *M. proteolyticum* species. The previously mentioned taxonomical 16S rDNA value has been equated to the species delineation threshold ANI values of 95–96% [DNA–DNA hybridization (DDH) value of 70%] (Richter and Rosselló-Móra, 2009). TL13 exhibited 82.4 and 75.2% ANI with *Microbacterium enclense* NIO-1002 and *Microbacterium hominis* TPW29, respectively, based on JSpeciesWS. Consequently, TL13 should not belong neither to *M. enclense* nor to *M. hominis* species. Accordingly, TL13 is a novel species within the genus *Microbacterium*, for which the name *M. metallidurans* is proposed. Figure 4 shows the graphical representation map of the genome of TL13 strain compared with the genomes of *M. enclense* NIO-1002 and *M. hominis* TPW29. The complete genome of *M*. *metallidurans* TL13 measured 3,587,460 bp with 70.70% G + C content (Table 3). *De novo* assembly of TL13 strain genome sequencing data is represented by 5 contigs and 3,393 coding sequences.

**Table 3:** Characteristics of *Microbacterium metallidurans* TL13 genome.

**Genome**	***Microbacterium metallidurans* TL13**
Domain	Bacteria
Taxonomy	Bacteria; *Terrabacteria* group; *Actinobacteria*; *Actinobacteria*; *Micrococcales*; *Microbacteriaceae*; *Microbacterium*; *Microbacterium metallidurans* TL13
Size	3,587,460
GC Content	70.7
N50	1,413,381
L50	1
Number of Contigs (with PEGs)	5
Number of Subsystems	380
Number of Coding Sequences	3,393
Number of RNAs	52

Based on the annotation of the RAST pipeline, TL13 genes were classified into 380 subsystems revealing its high metabolic diversity. Analysis of the predicted protein sequences showed that most of the annotated genes were involved in carbohydrates (399), amino acids and their derivatives (354), cofactors, vitamins, prosthetic groups, pigments (214), protein metabolism (217), fatty acids, lipids and isoprenoids (129), cell membrane and capsule (91), DNA (88) and RNA (85) metabolism and response to stresses (74), virulence, defense (55), respiration (55), metabolism of aromatic compounds (47), and phosphorus metabolism (40).

Analysis of *M*. *metallidurans* TL13 genome revealed the presence of several genes contributing directly or indirectly to PGP activities (Table 4). Indeed, the annotation study showed the presence of genes involved in the solubilization of inorganic phosphate, assimilation of ammonia, production of levan (EPS), synthesis of IAA, siderophores and polyketides, as well as genes encoding the key cell-wall-degrading enzymes (Table 4).

**Table 4:** Rapid Annotations using Subsystems Technology (RAST) predicted proteins potentially associated with plant growth promotion traits within the genome of *Microbacterium metallidurans* TL13.

**Protein name**	**Start**	**Stop**	**Length (pb)**	**Feature ID**
**Ammonia assimilation**				
Ammonium transporter	28,180	26,909	1,272	fig|69370.7.peg.113
Glutamine synthetase type I (EC 6.3.1.2)	68,639	70,063	1,425	fig|69370.7.peg.148
Glutamate-ammonia-ligase adenylyltransferase (EC 2.7.7.42)	78,333	75,340	2,994	fig|69370.7.peg.153
Glutamine synthetase type I (EC 6.3.1.2)	79,681	78,341	1,341	fig|69370.7.peg.154
Glutamate synthase [NADPH] small chain (EC 1.4.1.13)	256,192	254,726	1,467	fig|69370.7.peg.2590
Glutamate synthase [NADPH] large chain (EC 1.4.1.13)	260,762	256,185	4,578	fig|69370.7.peg.2591
**Inorganic phosphate assimilation and solubilization**				
Probable low-affinity inorganic phosphate transporter	276,078	277,253	1,176	fig|69370.7.peg.349
	87,776	86,481	1,296	fig|69370.7.peg.2726
Phosphate regulon transcriptional regulatory protein PhoB (SphR)	695,530	696,507	978	fig|69370.7.peg.2090
	11,84,663	11,85,424	762	fig|69370.7.peg.1222
	750,067	749,384	684	fig|69370.7.peg.804
Phosphate ABC transporter, periplasmic phosphate-binding protein PstS (TC 3.A.1.7.1)	766,996	768,117	1,122	fig|69370.7.peg.821
	848,746	847,049	1,698	fig|69370.7.peg.902
	860,264	858,657	1,608	fig|69370.7.peg.910
Phosphate regulon sensor protein PhoR (SphS) (EC 2.7.13.3)	751,281	750,064	1,218	fig|69370.7.peg.805
Inorganic pyrophosphatase (EC 3.6.1.1)	941,934	942,467	534	fig|69370.7.peg.2325
Exopolyphosphatase (EC 3.6.1.11)	626,058	627,020	963	fig|69370.7.peg.2018
**Auxin synthesis**				
Tryptophan synthase alpha chain (EC 4.2.1.20) involved in auxin synthesis	262,621	261,830	792	fig|69370.7.peg.2593
Tryptophan synthase beta chain (EC 4.2.1.20) involved in auxin synthesis	263,952	262,618	1,335	fig|69370.7.peg.2594
Anthranilate phosphoribosyltransferase (EC 2.4.2.18)	68,751	69,812	1,062	fig|69370.7.peg.2400
phosphoribosylanthranilate isomerase (EC 5.3.1.24)	262,621	261,830	792	fig|69370.7.peg.2593
Monoamine oxidase (1.4.3.4)	885,447	887,435	1,989	fig|69370.7.peg.931
	833,234	831,207	2,028	fig|69370.7.peg.2215
**Siderophore synthesis**				
Siderophore -interacting protein	179,305	180,231	927	fig|69370.7.peg.2511
ABC-type Fe^3+^-siderophore transport system, permease 2 component	777,693	778,700	1,008	fig|69370.7.peg.832
ABC-type Fe^3+^-siderophore transport system, ATPase component	181,137	180,313	8,25	fig|69370.7.peg.2512
ABC-type Fe^3+^-siderophore transport system, permease 2 component	182,192	181,134	1,059	fig|69370.7.peg.462
ABC-type Fe^3+^-siderophore transport system, permease component	183,235	182,189	1,047	fig|69370.7.peg.661
Putative ABC iron siderophore transporter, fused permease and ATPase domains	405,489	403,672	1,818	fig|69370.7.peg.3014
ABC-type Fe^3+^-siderophore transport system, permease component	776,716	777,696	981	fig|69370.7.peg.831
Putative ABC iron siderophore transporter, fused permease and ATPase domains	587,004	587,765	762	fig|69370.7.peg.647
**Potent fungal cell-wall-degrading enzymes**				
Carboxymethyl cellulase (EC 3.2.1.4)	694,440	695,393	954	fig|69370.7.peg.868
Serine protease, subtilase family (EC 3.4.21.-)	19,362	15,790	3,573	fig|69370.7.peg.1452
Putative serine protease	480,754	479,579	1,176	fig|69370.7.peg.534
β-hexosaminidase (EC 3.2.1.52)	602,405	603,559	1,155	fig|69370.7.peg.3342
	103,467	103,277	1,881	fig|69370.7.peg.534
**Exopolysaccharide synthesis**				
Levansucrase (EC 2.4.1.10)	646,212	647,819	1,608	fig|69370.7.peg.1075
**Polyketide Synthesis**				
Regulator of polyketide synthase expression	95,550	96,821	1,272	fig|69370.7.peg.171
Malonyl CoA-acyl carrier protein transacylase (EC 2.3.1.39)	96,904	97,824	921	fig|69370.7.peg.172
3-oxoacyl-[acyl-carrier-protein] synthase, KASIII (EC 2.3.1.41)	97,821	98,825	1,005	fig|69370.7.peg.173
Acyl carrier protein	98,900	99,148	249	fig|69370.7.peg.174
3-oxoacyl-[acyl-carrier-protein] synthase, KASII (EC 2.3.1.179)	99,214	100,452	1,239	fig|69370.7.peg.175
Chalcone synthase (EC 2.3.1.74)	912,875	913,969	1,095	fig|69370.7.peg.956

**Figure 5:**
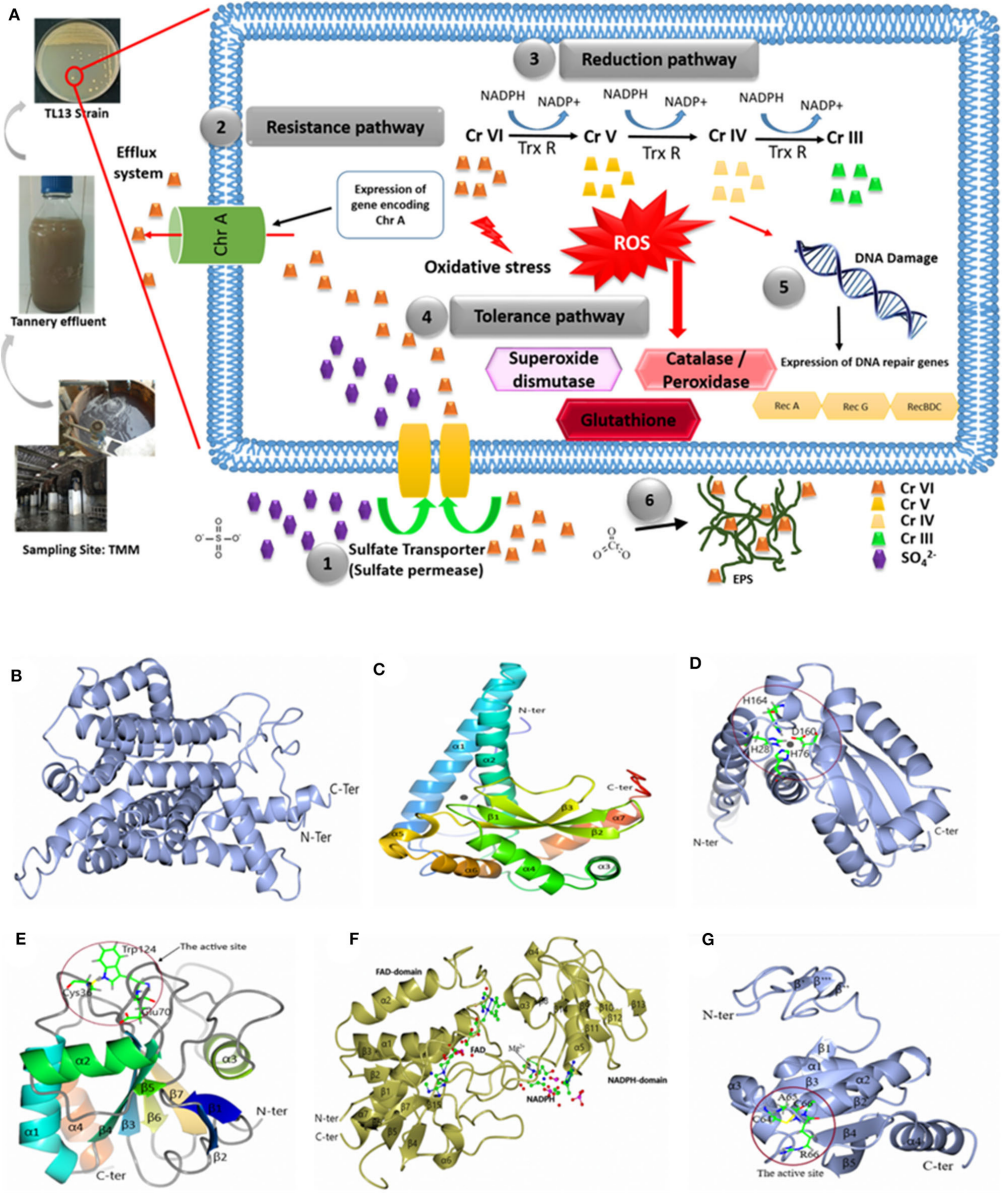
**(A)** Hypothetical pathways involved in chromium resistance and reduction in *M. metallidurans* TL13: (1) The sulfate permease allows chromium to get into bacterial cell; (2) expression of gene encoding chromate transporter ChrA (Efflux system); (3) reduction of chromium due to thioredoxin reductase and NADPH; (4) oxidative stress due to chromium reduction [Cr(V) and Cr(IV)] induced reactive oxygen species (ROS): superoxide dismutase, catalase, peroxidase, and glutathione; (5) DNA damage: expression of dna repair genes (RecA, RecG, and RecBDC); and (6) exopolysaccharides (EPS) and metal complex. Cartoon representation of 3D structure of TL13: **(B)** chromate transporter; **(C)** superoxide dismutase; **(D)** the active site of superoxide dismutase; **(E)** glutathione peroxidase with the localization of the active site; **(F)** thioredoxin reductase with the localization of reduced nicotinamide adenine dinucleotide phosphate (NADPH) and flavin adenine dinucleotide (FAD) domain, and **(G)** 3D structure of TL13 thioredoxin with the localization of the active site.

The genome of *M. metallidurans* TL13 contains 32 genes related to heavy metal resistance. Based on genomic analyses, enzymatic and non-enzymatic mechanisms could be proposed to explain hexavalent chromium resistance and removal by this strain (Figure 5).

The first chromium resistance mechanism to mention is the chromate transporter protein (ChrA) encoding by fig|69370.7.peg.2330 gene and localized in contig 29. The predicted model for ChrA indicated that the enzyme is formed by 19 α-helices: α1 (Gly24–Val37), α2 (Asp45–Ala62), α3 (Ser64–Ala75), α4 (Phe77–Thr88), α5 (Ser91–Ala97), α6 (Gly101–Leu104), α7 (Gly107–Gly 131), α8 (Asp139–Ala159), α9 (Gly164–Ala180), α10 (Gly192–Leu209), α11 (Ala227–Glu251), α12 (Ser259–Val273), α13 (Pro276–Gly285), α14 (Val295–Val303), α15 (Leu307–Leu323), α16 (Gly335–Leu348), α17 (Pro360–Phe363), α18 (Ala366–Phe376), and α19 (Val384–Leu396) (Figure 5B).

The second gene potentially involved in the bacterial chromium reduction by TL13 strain is the superoxide dismutase with a molecular weight of 23,177.87 Da, encoded by the gene fig|69370.7.peg.2482. The superoxide dismutase predicted model indicated that this enzyme belongs to the metalloenzyme family (EC1.15.1.1), which is composed of seven α-helices (α1, Thr23–Lys53; α2, Arg61–Thr81; α3, Asp94–Asp100; α4, Asp107–Gly119; α5, His164–Tyr170; α6, Ala175–Asn183; and α7, Asp 189–Arg198), three-stranded antiparallel β sheets (β1, Gly124–Asp131; β2, Asn136–Phe143; and β3, Val154–Asp160), and loops joining these secondary structure elements. The monomer structure of the enzyme contains two domains: the rod-shaped N-terminal domain (N-terminal extended region and two α-helices) and the globular (α + β)-type C-terminal domain (Figure 5C). The active site is composed of His28, His76, Asp160, and His164 (Figure 5D).

According to genome analyses, the glutathione peroxidase could also be involved in chromium reduction. This enzyme, with a molecular weight of 17,787.16 Da and an isoelectric point of 5.66, is encoded by the gene fig|69370.7.peg.2340. The gene is localized on the contig 4. The predicted model for glutathione peroxidase indicated that its structure is composed of seven β strands (β1, Pro7–Thr10; β2, Gly13–Thr17; β3, Ala26–Val32; β4, Phe57–Pro63; β5, Met91–Ser92; β6, Lys128–Phe132; and β7, Gly135–Phe140), four α-helices (α1, Gln41–Arg55; α2, Glu74–Trp85; α3, Pro104–Thr112; and α4, Pro149–Pro160), and loops joining these elements together. The structural analysis demonstrated that (i) the N-terminal end is formed by two β strands (β1 and β2), (ii) the enzyme contains central twisted β sheets (β3, β4, β5, β6, and β7) boarded by helices α1, α2, and α4 on one side and helix α3 on the other side, and (iii) the C-terminal end is composed of helix α4, which is nearly parallel with the α1. The model contains 161 residues from Met1 to His161, and the amino acid residues of the catalytic triad are Cys36, Gln70, and Trp124 (Figure 5E).

The TL13 genome analysis revealed also the presence of three genes encoding thioredoxin reductases (fig|69370.7.peg.1744 and fig|69370.7.peg.1847 localized on the contig 29 and fig|69370.7.peg.2708 localized on the contig 45). The predicted model for the thioredoxin reductase encoded by gene (fig|69370.7.peg.1847) indicated that the structure is composed of seven α-helix α1(Gly10–Met23) α2 (Leu57–Ala67), α3 (Ala119–Arg124), α4 (Gly134–Phe137), α5 (Pro149–Val161), α6 (Phe210–214), and α7 (Leu256–Pro277) and 15 β strands β1 (Ala4–Ile6), β2 (Thr27–Leu29), β3 (Val71–Ile73), β4 (Ser79–Ala82), β5 (Val88–Val92), β6 (Glu 95–Ala99), β7 (Tyr101–Leu104), β8 (Val126–Ala127), β9 (Ala143–Val144), β10 (Val166–Val167), β11 (Val173–Arg175), β12 (Thr186–Leu187), β13 (Gly190–Thr192), β14 (Ala198–Phe199), and β15 (Leu238–Ala239). The structure is composed of the flavin adenine dinucleotide (FAD)-binding domain (Ser32, Glu 34, Tyr35, Val40, Ala43, Val 78, Thr106, Gly 107, Cys 132, Arg 207, Thr 255, Leu 256) and the reduced nicotinamide adenine dinucleotide phosphate (NADPH)-binding domain (Pro114, Ala147, Ser148, His 150, Lys153, Val203, His 248) (Figure 5F). The predicted model of the thioredoxin indicated that the N-terminal domain is composed of three β strands β′ (Ala17–Val18), β″ (Arg25–Arg26), and β″′ (Gln28–Gly29). The central structure is composed of five β strands [β1 (Glu38–Ileu39), β2 (Thr55–Ser60), β3 (Arg85–Asp90), β4 (Thr108–Asp113), and β5 (Gly116–Phe122)] surrounded by four α-helices (α1 (Pro42–Leu50), α2 (Arg66–Asp80), α3 (Leu91–His100), and α4 (Gly127–Ala143). It displays a strictly conserved CXXC catalytic active-site motif (Figure 5G). In addition to the abovementioned mechanisms, *M. metallidurans* TL13 can protect itself from the infiltration of toxic chromium ions by covering its peripheral surface with a shield of levan, a fructose homopolysaccharide, which can be synthesized by levan sucrase (fig|69370.7.peg.1075). Bioaccumulation might represent a supplementary route to achieve chromium resistance by TL13.

## Discussion

Bacteria of the genus *Microbacterium* (phylum of Actinobacteria) include more than 110 cultivable species (http://www.bacterio.net/microbacterium.html) isolated from aquatic and terrestrial ecosystems and from food and clinical samples. Figure S3 illustrates the microbial world (according to the Gold and DSMZ databases) with each point representing a type of bacterial strain, and if applicable, the associated ongoing sequencing projects (colored red). As shown in Figure S3, many taxonomic groups still need to be explored at the genomic level and, in particular, the *Microbacterium* group for which only few genomes are available (https://microbial-earth.namesforlife.com/). Several *Microbacterium* members have been isolated from extreme environments (Li et al., 2008) or contaminated with organic pollutants (Schippers et al., 2005) and metals such as uranium (Reardon et al., 2004; Gallois et al., 2018), plutonium and iron(III) (John et al., 2001), arsenic (Cai et al., 2009), and chromium (Pattanapipitpaisal et al., 2001; Humphries et al., 2005).

There are only few studies reported in the literature on the predominance and ecological importance of bacterial populations of the genus *Microbacterium* as PGPB (Alves et al., 2015; Gao et al., 2017). Although such bacteria directly stimulate plant growth by increasing soil nutrient uptake, inducing and producing plant growth regulators, and activating induced resistance mechanisms in plants, special attention has recently been given to bioremediation of organic and inorganic pollutants by PGPB (Bishnoi, 2015). Therefore, the present study aimed to characterize a new heavy-metal-resistant PGPB isolated from a Tunisian tannery wastewater for potential application as a green and sustainable tool for decreasing accumulation of metals in industrial sludges and wastewaters and in plant rhizosphere and tissues.

The actinobacterium TL13 exhibited resistance to all of the tested heavy metal ions. The half inhibitory concentration values of mercury, cobalt, chromium, copper, nickel, iron, and arsenic were 368, 445, 676, 1,590, 1,680, 4,403, and 7,007 mg/L, respectively, and their toxic order was HgCl_2_ > CoCl_2_ > K_2_Cr_2_O_7_ > CuSO_4_ > NiCl_2_ > FeSO_4_ > Na_2_HAsO_4_. TL13 possesses *in vitro* PGP traits including nitrogen fixation and inorganic phosphate solubilisation as well the production of PGP metabolites like IAA, siderophore, EPS, and cellulases. The growth-promoting capacity of TL13 strain was confirmed *in vivo* by measuring shoots length and fresh and dry weight of tomato seedlings in the presence of increasing chromium concentrations. Inoculation of TL13 to tomato increased plant growth by 22.77% of shoot length, 12.79% of fresh weight, and 34.62% of dry weight.

The whole genome of TL13 strain was sequenced and annotated in order to provide information on genomic elements related to heavy metal resistance and plant growth-promoting traits. The genome consists of 3,587,460 bp with an average GC content of 70.7% and a total of 52 genes encoded RNAs. Of the 3,393 genes predicted, 2,425 were identified as genes encoding proteins (genes had a specific function) and 968 genes (28.53%) were classified as hypothetical proteins (genes without function prediction). The ANI values between the strain TL13 and the closely related strains were 76.5–82.7%, indicating that TL13 strain is a novel species of the genus *Microbacterium*.

Functional annotation of TL13 genome revealed the presence of genes encoding proteins involved in resistance to metal stress, such as cobalt–zinc–cadmium resistance protein, copper resistance protein, copper responsive transcriptional regulator, arsenical resistance operon repressor, arsenate reductase, arsenic resistance protein, mercuric resistance operon regulatory protein, mercuric ion reductase, and organomercurial lyase. The TL13 genome analysis revealed also the presence of different potential genes involved in chromium reduction such as chromate transporter, superoxide dismutase, glutathione peroxidase, and the thioredoxin reductase. The chromate transporter protein (ChrA) has been reported to play a crucial role in efflux of cytoplasmic chromate (Alvarez et al., 1999; Ahemad and Kibret, 2014). The participation of bacterial enzymes in the defense against oxidative stress induced by chromate represents an additional mechanism of chromate resistance (McCord and Fridovich, 1988; Ackerley et al., 2006; Ramirez-Diaz et al., 2008). The TL13 strain has the potential to have a powerful enzyme system to combat oxidative stress involving catalase (EC 1.11.1.6), glutathione peroxidase, and superoxide dismutase [Mn] (EC 1.15.1.1). The theriodixin reductase, an Mg^2+^-dependent enzyme, is also involved in the reduction in Cr(VI) (Li and Krumholz, 2009; Collet and Messens, 2010). The two cysteines of the catalytic motif (Cys64, A65, R66, Cys67), located at the amino end of the two-helix, are the key players involved in the reduction of oxidized substrates. Chen et al. (2014) reported that the entire operon of thioredoxin of *Streptomyces violaceoruber* strain LZ-26-1 was upregulated under the stress of Cr(VI). This result suggests that TL13 strain can use thioredoxin pathway to reduce Cr(VI). Various oxidoreductases have been shown to reduce Cr(VI) such as ChrR from *E. coli* and *Pseudomonas* and NfsA from *E. coli* (Ackerley et al., 2004). Genes coding for Cr(VI)-reducing oxidorecductase were not detected on TL13 genome. This result probably shed light on novel Cr(VI) reductases within the thioredoxin pathway.

*M. metallidurans* TL13 was able to produce levan based on genomic signature (presence of the gene encoding levan sucrase) linked to experimental evidence (mucoid phenotype on agar plate). The anionic property of the EPS enables it to trap the positively charged chromium by electrostatic interaction and hence playing a key role as biosorbents for metal remediation (Harish et al., 2012; Gupta and Diwan, 2017).

TL13 genome encodes an efficient framework for iron acquisition mediated by a siderophore-dependent pathway as similar to that of *Cupriavidus metallidurans* CH34 (Tables S2, S3). Iron is essential for vital processes including DNA, RNA, and protein synthesis, electron transport, cell respiration and proliferation, as well as gene expression regulation. Iron has the ability to easily acquire and lose electrons from the iron form Fe^2+^ to the iron form Fe^3+^, and vice versa. This rather unique property gives it an essential role in oxidation and reduction processes. The chemical properties of iron make it potentially toxic. When iron is in limited supply, TL13 can produce and secrete siderophores capable of chelating ferric Fe^3+^ with very high affinity. The siderophore–Fe^3+^ complexes are then recovered by the bacterium using specific membrane transporters. The dissociation of iron from its chelator generally requires a reduction in Fe^3+^ to Fe^2+^ and a structural modification of the siderophore, making it less affine for the metal (Payne et al., 2016; Li and Ma, 2017).

Besides these genes conferring resistance to metal and oxidative stresses identified in TL13 genome, the RAST analysis showed the presence of 10 genes involved in osmoadaptation, in particular in the biosynthesis of betaine and choline osmolytes, 14 proteins (operon *dnaK*) to fight against thermal stress, and 4 against cold (proteins from the CspA family). Analysis of the genome of *M. metallidurans* TL13 revealed also the presence of 118 genes involved in different pathways of central carbohydrate metabolism like the tricarboxylic acid cycle and the pentose phosphate pathway. It also indicated the presence of 43 proteins involved in anaerobic fermentation processes. The genome of *M. metallidurans* TL13 codes for exopolyphosphatase (EC 3.6.1.11), inorganic manganese-dependent pyrophosphatase (EC 3.6.1.1), and low affinity inorganic phosphate carriers, which play a crucial role in phosphorus cycle by solubilizing its inorganic form that is fixed and precipitated in the soil (Al-Kaisi et al., 2013; Bhattacharyya et al., 2017). Moreover, the genome of *M. metallidurans* TL13 encodes all the genes necessary for ammonia assimilation and plant auxin biosynthesis (Spaepen and Vanderleyden, 2011). Several *Microbacterium* strains isolated from rhizospheric and non-rhizospheric environments are able to fix nitrogen, as confirmed by acetylene-reducing activity and PCR amplification of *nifH* gene (Gtari et al., 2011). Whereas, some nitrogen-fixing bacteria are able to produce nitrogenases with iron as the only metal cofactor, other bacterial strains incorporate molybdenum or vanadium into the cofactor as well (Johnstone and Nolan, 2015). For the strain TL13, a search for nitrogenase systems merits further investigation at the genome and physiological levels.

Besides these direct PGP traits, several genes involved in the biocontrol of phytopathogens exist in the genome of *M. metallidurans* TL13, including genes coding for cell-wall-degrading enzymes (cellulose, β-hexosaminidase, and proteases) (Dutta and Thakur, 2017) and genes coding enzymes responsible for the biosynthesis of polyketides, a class of secondary metabolites with antimicrobial roles (Khanna et al., 2019). *M. metallidurans* TL13 showed resistance to β-lactams and fluoroquinolones antibiotics leading to improved chance of survival in a competitive rhizospheric soil (Bhattacharyya et al., 2017). All genomic plant growth-promoting features make TL13 strain an excellent actinobacterium for utilization in sustainable agriculture (Table 5). Overall, these PGP results were in line with those reported for *Microbacterium* sp. strain 3J1 (Manzanera et al., 2015), *Microbacterium* sp. Yaish 1 (Jana et al., 2017), and *Microbacterium hydrothermale* BPSAC84 (Passari et al., 2019b).

**Table 5:** Genomic features and biotechnological potential of *M. metallidurans* TL13 compared to other *Microbacterium* species.

**Strain**	**Origin**	**Genome length (Mb)**	**GC (%)**	**Pathways and genes of interest**	**Bioremediation potential**	**References**
*M. oleivorans strain Wellendorf*	Hydrocarbon-contaminated soil	2.92	69.57	4-hydroxyphenylacetate degradation; Nitronate detoxification	Environmental pollutants detoxification	Avramov et al., 2016
*Microbacterium* sp. A20	Heavy metal contaminated soil (Indiana, USA)	3.94	68.53	Co/Zn/Cd efflux system; Tolerance to antibiotics; Chromium reductase (chrR)	Reduction of Cr VI into Cr III; Tolerance of cobalt, cadmium, and nickel	Learman et al., 2019
*Microbacterium* sp. K19		3.89	68.69			
*Microbacterium* sp. K21		3.85	68.33			
*M. oxydans* BEL4b	Rhizosphere of *Brassica napus* (Belgium)	3.8	68.27	Heavy metals resistance; Production of terpenoids Production of polyketides	Promoting plant growth in heavy metals contaminated soils	Corretto et al., 2015
*M. Azadirachtae* ARN176	Heavy metals contaminated Soil (Austria)	3.91	70.14			
*M. laevaniformans* Strain OR221	Field Research Center (Tennessee, USA)	3.4	68	Heavy-metal transport proteins	Tolerance of heavy metals and acidic conditions	Brown et al., 2012
*M. testaceum* StLB037	Potato leaves (Japan)	3.98	70.28	Lactonases genes	Biocontrol agent against phytopathogens	Morohoshi et al., 2011
*M. profundi* Shh49	Sea sediment (Pacific Ocean)	3.36	66.54	Multicopper oxidases (MCOs); Mercuric reductase	Reduction of mercury in contaminated environments	Wu et al., 2015
*M. metallidurans* TL13	Tannery wastewater (Tunisia)	3.58	70.7	Genes involved in heavy metal resistance and plant growth promotion	Bioremediation of tannery wastewater and metal contaminated soil; Plant growth promotion under metallic stress	This work

## Conclusion

This study reports the characterization of a heavy-metal-resistant, plant growth-promoting actinobacterium, *M. metallidurans* TL13, isolated from a tannery effluent. Genome analyses, as well as experimental studies, indicated that *M. metallidurans* TL13 could promote plant growth, especially by solubilizing inorganic phosphate, synthesizing siderophores, and producing IAA, hydrolytic enzymes, and EPS. Besides, genome annotation revealed the presence of multiple genetic determinants related to the resistance to heavy metals, particularly chromium, arsenic, and mercury. To the best of our knowledge, this is the first report providing new insights into the molecular mechanisms underlying the enhancement of plant growth and the resistance to chromium and other heavy metals in a new species of *Microbacterium*. Further comparative genomic analysis and direct redox experimentation will provide additional information about the variance and pathways involved in the multimetal resistance and rhizosphere bioremediation by the actinobacterium *M. metallidurans* TL13 that is expected to facilitate environmental and agricultural applications in heavy metal-polluted sites.

## Data Availability Statement

The Whole Genome Shotgun project has been deposited at DDBJ/ENA/GenBank under the accession SZZQ00000000. The version reported in this work is SZZQ01000000.

## Author Contributions

MN, RO, HCho, and AChe conceived and designed the experiments. HChe, YB, and RO performed PGP experiments. ACha and HK performed chemical analysis. AN, HS, RO, AM, MM, and MN performed bioinformatics and structural analysis. HK contributed reagents, material tools. MN, RO, AO, AN, HChe, MM, AM, HS, HCho, and AChe analyzed the data. RO, AO, MM, AN, MN, HS, HChe, and AChe prepared and revised the manuscript. MN and AChe supervised the entire project. All authors contributed to the article and approved the submitted version.

## Conflict of Interest

The authors declare that the research was conducted in the absence of any commercial or financial relationships that could be construed as a potential conflict of interest.

## References

[B1] Ackerley D. F. Barak Y. Lynch S. V. Curtin J. Matin A. (2006). Effect of chromate stress on *Escherichia coli* K-12. J. Bacteriol. 188, 3371–3381. 10.1128/JB.188.9.3371-3381.2006 16621832 PMC1447458

[B2] Ackerley D. F. Gonzalez C. F. Keyhan M. Blake I. R. Matin A. (2004). Mechanism of chromate reduction by the *Escherichia coli* protein, NfsA, and the role of different chromate reductases in minimizing oxidative stress during chromate reduction. Environ. Microbiol. 6, 851–860. 10.1111/j.1462-2920.2004.00639.x 15250887

[B3] Ahemad M. Kibret M. (2014). Mechanisms and applications of plant growth promoting rhizobacteria: current perspective. J. King. Saud. Univ. Sci. 26, 1–20. 10.1016/j.jksus.2013.05.001

[B4] Alexander D. B. Zuberer D. A. (1991). Use of chrome azurol S reagents to evaluate siderophore production by rhizosphere bacteria. Biol. Fert. Soil. 12, 39–45. 10.1007/BF00369386

[B5] Al-Kaisi M. M. Elmore R. W. Guzman J. G. Hanna H. M. Hart C. E. Helmers M. J. (2013). Drought impact on crop production and the soil environment: 2012 experiences from Iowa. J. Soil. Water. Conserv. 68, 19–24. 10.2489/jswc.68.1.19A

[B6] Alvarez A. H. Moreno-Sánchez R. Cervantes C. (1999). Chromate efflux by means of the ChrA chromate resistance protein from *Pseudomonas aeruginosa*. J. Bacteriol. 181, 7398–7400. 10.1128/JB.181.23.7398-7400.1999 10572148 PMC103707

[B7] Alves A. Riesco R. Correia A. Trujillo M. E. (2015). *Microbacterium proteolyticum* sp. nov. isolated from roots of Halimione portulacoides. Int. J. Syst. Evol. Microbiol. 65, 1794–1798. 10.1099/ijs.0.000177 25744585

[B8] Ashraf S. Afzal M. Naveed M. Shahid M. Zahir Z. A. (2018). Endophytic bacteria enhance remediation of tannery effluent in constructed wetlands vegetated with Leptochloa fusca. Int. J. Phytoremediat. 20, 121–128. 10.1080/15226514.2017.1337072 28621547

[B9] Avramov A. P. Couger M. B. Hartley E. L. Land C. Wellendorf R. Hanafy R. A.. (2016). Draft genome sequence of *Microbacterium oleivorans* strain Wellendorf implicates heterotrophic versatility and bioremediation potential. Genom. Data. 10, 54–60. 10.1016/j.gdata.2016.09.005 27699150 PMC5035333

[B10] Aziz R. K. Bartels D. Best A. A. DeJongh M. Disz T. Edwards R. A.. (2008). The RAST server: rapid annotations using subsystems technology. BMC Genomics 9:75. 10.1186/1471-2164-9-75 18261238 PMC2265698

[B11] Baldiris R. Acosta-Tapia N. Montes A. Hernández J. Vivas-Reyes R. (2018). Reduction of hexavalent chromium and detection of Chromate Reductase (ChrR) in Stenotrophomonas maltophilia. Molecules 23:406. 10.3390/molecules23020406 29438314 PMC6017488

[B12] Balmer J. (2018). Hexavalent chromium. Workplace Health Saf. 66:564. 10.1177/2165079918805775 30340452

[B13] Bankevich A. Nurk S. Antipov D. Gurevich A. A. Dvorkin M. Kulikov A. S. (2012). SPAdes: a new genome assembly algorithm and its applications to single-cell sequencing. J. Comp. Biol.19, 455–477. 10.1089/cmb.2012.0021 PMC3342519 22506599

[B14] Bauer A. W. Kirby W. M. Sherris J. C. Turck M. (1966). Antibiotic susceptibility testing by a standardized single disk method. Am. J. Clin. Pathol. 45, 493–496. 5325707

[B15] Bharagava R. N. Mishra S. (2018). Hexavalent chromium reduction potential of *Cellulosimicrobium* sp. isolated from common effluent treatment plant of tannery industries. Ecotox. Environ. Saf. 147, 102–109. 10.1016/j.ecoenv.2017.08.040 28841524

[B16] Bhattacharyya C. Bakshi U. Mallick I. Mukherji S. Bera B. Ghosh A. (2017). Genome-guided insights into the plant growth promotion capabilities of the physiologically versatile bacillus aryabhattai strain AB211. Front. Microbiol. 8:411 10.3389/fmicb.2017.00411 28377746 PMC5359284

[B17] Bishnoi U. (2015). “Chapter four - PGPR interaction: an ecofriendly approach promoting the sustainable agriculture system,” in Advances Botanical Research, eds H. Bais and J. Sherrier (Newark, DE: Academic Press), 81–113.

[B18] Brown S. D. Palumbo A. V. Panikov N. Ariyawansa T. Klingeman D. M. Johnson C. M.. (2012). Draft genome sequence for *Microbacterium laevaniformans* strain OR221, a bacterium tolerant to metals, nitrate, and low pH. J. Bacteriol. 194, 3279–3280. 10.1128/JB.00474-12 22628508 PMC3370866

[B19] Cai T. Ju S. Lee J. Sai N. Demkov A. A. Niu Q. (2009). Magnetoelectric coupling and electric control of magnetization in ferromagnet/ferroelectric/normal-metal superlattices. Phys. Rev. B. 80:140415 10.1103/PhysRevB.80.140415

[B20] Chai L. Ding C. Li J. Yang Z. Shi Y. (2019). Multi-omics response of *Pannonibacter phragmitetus* BB to hexavalent chromium. Environ. Pollut. 249, 63–73. 10.1016/j.envpol.2019.03.005 30878863

[B21] Chen H. Yin Y. Feng E. Li Y. Xie X. Wang Z. (2014). Thioredoxin peroxidase gene is involved in resistance to biocontrol fungus *Nomuraea rileyi* in *Spodoptera litura*: gene cloning, expression, localization and function. Dev. Comp. Immunol. 44, 76–85. 10.1016/j.dci.2013.11.012 24296440

[B22] Collet J. F. Messens J. (2010). Structure, function, and mechanism of thioredoxin proteins. Antioxid. Redox. Signal. 13, 1205–1216. 10.1089/ars.2010.3114 20136512

[B23] Corretto E. Antonielli L. Sessitsch A. Kidd P. Weyens N. Brader G. (2015). Draft genome sequences of 10 *Microbacterium* spp., with emphasis on heavy metal-contaminated environments. Genome. Announc. 3:e00432–e00415. 10.1128/genomeA.00432-15 25977426 PMC4432332

[B24] Davis I. W. Leaver-Fay A. Chen V. B. Block J. N. Kapral G. J. Wang X. (2007). MolProbity: all-atom contacts and structure validation for proteins and nucleic acids. Nucleic. Acids. Res. 35(Suppl. 2), W375–W383. 10.1093/nar/gkm216 17452350 PMC1933162

[B25] Davis S. R. (2005). An overview of the antifungal properties of allicin and its breakdown products - the possibility of a safe and effective antifungal prophylactic. Mycoses. 48, 95–100. 10.1111/j.1439-0507.2004.01076.x 15743425

[B26] Dutta J. Thakur D. (2017). Evaluation of multifarious plant growth promoting traits, antagonistic potential and phylogenetic affiliation of rhizobacteria associated with commercial tea plants grown in Darjeeling, India. PLoS ONE 12:e0182302 10.1371/journal.pone.0182302 28771547 PMC5542436

[B27] Elahi A. Ajaz M. Rehman A. Vuilleumier S. Khan Z. Hussain S. Z. (2019). Isolation, characterization, and multiple heavy metal-resistant and hexavalent chromium-reducing *Microbacterium* testaceum B-HS2 from tannery effluent. J. King. Saud. Univ. Sci. 31, 1437–1444. 10.1016/j.jksus.2019.02.007

[B28] Fang Z. Yong Y. C. Zhang J. Du G. Chen J. (2017). Keratinolytic protease: a green biocatalyst for leather industry. Appl. Microbiol. Biotechnol. 101, 7771–7779. 10.1007/s00253-017-8484-1 28924866

[B29] Fassy J. Tsalkitzi K. Salavagione E. Hamouda-Tekaya N. Braud V. M. (2017). A real-time digital bio-imaging system to quantify cellular cytotoxicity as an alternative to the standard chromium-51 release assay. Immunology. 150, 489–94. 10.1111/imm.12702 28004383 PMC5343348

[B30] Fu J. Lv H. Chen F (2016). Diversity and Variation of Bacterial Community Revealed by MiSeq Sequencing in Chinese Dark Teas. PLoS One. 11:e0162719 10.1371/journal.pone.0162719 27690376 PMC5045175

[B31] Gallois N. Piette L. Ortet P. Bakarat M. Long J. Berthomieu C.. (2018). Proteomics data for characterizing *Microbacterium oleivorans* A9, an uranium-tolerant actinobacterium isolated near the Chernobyl nuclear power plant. Data Brief 21, 1125–1129. 10.1016/j.dib.2018.10.136 30456224 PMC6231083

[B32] Galperin M. Y. Makarova K. S. Wolf Y. I. Koonin E. V. (2015). Expanded microbial genome coverage and improved protein family annotation in the COG database. Nucleic Acids Res. 43, D261–D269. 10.1093/nar/gku1223 25428365 PMC4383993

[B33] Gao J. L. Sun P. Wang X. M. Lv F. Y. Sun J. G. (2017). *Microbacterium* zeae sp. nov., an endophytic bacterium isolated from maize stem. Antonie Van Leeuwenhoek 110, 697–704. 10.1007/s10482-017-0837-3 28176143

[B34] Grant J. R. Stothard P. (2008). The CGView Server: a comparative genomics tool for circular genomes. Nucleic. Acids. Res. 36, W181–W84. 10.1093/nar/gkn179 18411202 PMC2447734

[B35] Gtari M. Ghodhbane-Gtari F. Nouioui I. Beauchemin N. Tisa L. S. (2011). Phylogenetic perspectives of nitrogen-fixing actinobacteria. Arch. Microbiol. 194, 3–11. 10.1007/s00203-011-0733-6 21779790

[B36] Gupta P. Diwan B. (2017). Bacterial Exopolysaccharide mediated heavy metal removal: a review on biosynthesis, mechanism and remediation strategies. Biotechnol. Rep. 13, 58–71. 10.1016/j.btre.2016.12.006 PMC5361134 28352564

[B37] Gutiérrez J. C. Amaro F. Martín-González A. (2015). Heavy metal whole-cell biosensors using eukaryotic microorganisms: an updated critical review. Front. Microbiol. 6:48 10.3389/fmicb.2015.00048 25750637 PMC4335268

[B38] Harish R. Samuel J. Mishra R. Chandrasekaran N. Mukherjee A. (2012). Bio-reduction of Cr(VI) by exopolysaccharides (EPS) from indigenous bacterial species of Sukinda chromite mine, India. Biodegradation 23, 487–496. 10.1007/s10532-011-9527-4 22119897

[B39] Hassen W. Neifar M. Cherif H. Najjari A. Chouchane H. Driouich R. C.. (2018). *Pseudomonas rhizophila* S211, a new plant growth-promoting rhizobacterium with potential in pesticide-bioremediation. Front. Microbiol. 9:34. 10.3389/fmicb.2018.00034 29527191 PMC5829100

[B40] Henson M. W. Santo Domingo J. W. Kourtev P. S. Jensen R. V. Dunn J. A. Learman D. R. (2015). Metabolic and genomic analysis elucidates strain-level variation in *Microbacterium* spp. isolated from chromate contaminated sediment. PeerJ. 3:e1395 10.7717/peerj.1395 26587353 PMC4647564

[B41] Humphries A. C. Nott K. P. Hall L. D. Macaskie L. E. (2005). Reduction of Cr(VI) by immobilized cells of *Desulfovibrio vulgaris* NCIMB 8303 and *Microbacterium* sp. NCIMB 13776. Biotechnol. Bioeng. 90, 589–596. 10.1002/bit.20450 15818565

[B42] Ibrahim A. S. S. El-Tayeb M. A. Elbadawi Y. B. Al-Salamah A. A. Antranikian G. (2012). Hexavalent chromate reduction by alkaliphilic *Amphibacillus* sp. KSUCr3 is mediated by copper-dependent membrane-associated Cr(VI) reductase. Extremophiles 16, 659–668. 10.1007/s00792-012-0464-x 22669507

[B43] Jakov B. (2005). “Costs of tannery waste treatment,” in 15th Session of the UNIDO Leather and Leather Products Industry Panel (Leon).

[B44] Jana G. A. Al-Yahyai R. Yaish M. W. (2017). Genome sequencing of *Microbacterium* sp. Yaish 1, a bacterial strain isolated from the rhizosphere of date palm trees affected by salinity. Genome Announc. 5:e01247-17 10.1128/genomeA.01247-17 29097476 PMC5668552

[B45] Jensen H. L. (1942). Nitrogen fixation in leguminous plants. I. General characters of root-nodule bacteria isolated from species of Medicago and Trifolium in Australia. P. Linn. Soc. N. S. W. 67, 98–108.

[B46] John S. G. Ruggiero C. E. Hersman L. E. Tung C. S. Neu M. P. (2001). Siderophore mediated plutonium accumulation by *Microbacterium flavescens* (JG-9). Environ. Sci. Technol. 35, 2942–2948. 10.1021/es010590g 11478246

[B47] Johnstone T. C. Nolan E. M. (2015). Beyond iron: Non-classical biological functions of bacterial siderophores. Dalton Trans. 44, 6320–6339. 10.1039/C4DT03559C 25764171 PMC4375017

[B48] Kang S. M. Radhakrishnan R. Khan A. L. Kim M. J. Park J. M. Kim B. R. (2014). Gibberellin secreting rhizobacterium, *Pseudomonas putida* H-2-3 modulates the hormonal and stress physiology of soybean to improve the plant growth under saline and drought conditions. Plant. Physiol. Biochem. 84, 115–124. 10.1016/j.plaphy.2014.09.001 25270162

[B49] Khanna K. Jamwal V. L. Sharma A. Gandhi S. G. Ohri P. Bhardwaj R. (2019). Supplementation with plant growth promoting rhizobacteria (PGPR) alleviates Cadmium toxicity in *Solanum lycopersicum* by modulating the expression of secondary metabolites. Chemosphere 230, 628–639. 10.1016/j.chemosphere.2019.05.072 31128509

[B50] Kim M. Oh H. S. Park S. C. Chun J. (2014). Towards a taxonomic coherence between average nucleotide identity and 16S rRNA gene sequence similarity for species demarcation of prokaryotes. Int. J. Syst. Evol. Microbiol. 64, 346–351. 10.1099/ijs.0.059774-0 24505072

[B51] Kim O. Cho Y. Lee K. Yoon S. Kim M. Na H.. (2012). Introducing EzTaxon-e: a prokaryotic 16S rRNA gene sequence database with phylotypes that represent uncultured species. Int. J. Syst. Evol. Microbiol. 62, 716–721. 10.1099/ijs.0.038075-0 22140171

[B52] Kumar M. Saini H. S. (2019). Reduction of hexavalent chromium (VI) by indigenous alkaliphilic and halotolerant *Microbacterium* sp. M5: comparative studies under growth and nongrowth conditions. J. Appl. Microbiol. 127, 1057–1068. 10.1111/jam.14366 31260173

[B53] Kumar S. Stecher G. Tamura K. (2016). MEGA7: molecular evolutionary genetics analysis version 7.0 for bigger datasets. Mol. Biol. Evol. 33, 1870–1874. 10.1093/molbev/msw054 27004904 PMC8210823

[B54] Learman D. R. Ahmad Z. Brookshier A. Henson M. W. Hewitt V. Lis A.. (2019). Comparative genomics of 16 *Microbacterium* spp. that tolerate multiple heavy metals and antibiotics. PeerJ. 6:e6258. 10.7717/peerj.6258 30671291 PMC6336093

[B55] Learman D. R. Voelker B. M. Vazquez-Rodriguez A. I. Hansel C. M. (2011). Formation of manganese oxides by bacterially generated superoxide. Nat. Geosci. 4, 95–98. 10.1038/ngeo105524027565

[B56] Li H. Li Z. Liu T. Xiao X. Peng Z. Deng L. (2008). A novel technology for biosorption and recovery hexavalent chromium in wastewater by bio-functional magnetic beads. Bioresour. Technol. 99, 6271–6279. 10.1016/j.biortech.2007.12.002 18221868

[B57] Li X. Krumholz L. R. (2009). Thioredoxin is involved in U(VI) and Cr(VI) reduction in *Desulfovibrio desulfuricans* G20. J. Bacteriol. 191, 4924–4933. 10.1128/JB.00197-09 19482922 PMC2715717

[B58] Li Y. Ma Q. (2017). Iron acquisition strategies of vibrio anguillarum. Front. Cell. Infect. Microbiol. 7:342 10.3389/fcimb.2017.00342 28791260 PMC5524678

[B59] Lun L. Li D. Yin Y. Li D. Xu G. Zhao Z. (2016). Characterization of chromium waste form based on biocementation by *Microbacterium* sp. GM-1. Indian. J. Microbiol. 56, 353–60. 10.1007/s12088-016-0579-3 PMC4920760 27407300

[B60] Mahjoubi M. Aliyu H. Cappello S. Neifar M. Souissi Y. Cowan D. A. (2019). The genome of *Alcaligenes aquatilis* strain BU33N: insights into hydrocarbon degradation capacity. PLoS ONE 14:e0221574 10.1371/journal.pone.0221574 31550268 PMC6759156

[B61] Manzanera M. García-Fontana C. Vílchez J. I. Narváez-Reinaldo J. J. González-López J. (2015). Genome sequence of *Microbacterium* sp. strain 3J1, a highly desiccation tolerant bacterium that promotes plant growth. Genome Announc. 3:e00713-15. 10.1128/genomeA.00713-15 26316631 PMC4551875

[B62] Maqbool Z. Asghar H. N. Shahzad T. Hussain S. Riaz M. Ali S. (2015). Isolating, screening and applying chromium reducing bacteria to promote growth and yield of okra (*Hibiscus esculentus* L.) in chromium contaminated soils. Ecotox. Environ. Saf. 114, 343–349. 10.1016/j.ecoenv.2014.07.007 25066609

[B63] Markowitz V. M. Mavromatis K. Ivanova N. N. Chen I.-M. A. Chu K. Kyrpides N. C. (2009). IMG ER: a system for microbial genome annotation expert review and curation. Bioinformatics 25, 2271–2278. 10.1093/bioinformatics/btp393 19561336

[B64] McCord J. M. Fridovich I. (1988). Superoxide dismutase: The first twenty years (1968–1988). Free. Radic. Biol. Med. 5, 363–369. 10.1016/0891-5849(88)90109-8 2855736

[B65] McNicholas S. Potterton E. Wilson K. S. Noble M. E. M. (2011). Presenting your structures: the CCP4mg molecular-graphics software. Acta Crystallogr. D Biol. Crystallogr. 67, 386–394. 10.1107/S0907444911007281 21460457 PMC3069754

[B66] Morohoshi T. Wang W. Z. Someya N. Ikeda T. (2011). Genome sequence of *Microbacterium* testaceum StLB037, an N-acylhomoserine lactone-degrading bacterium isolated from potato leaves. J. Bacteriol. 193, 2072–2073. 10.1128/JB.00180-11 21357489 PMC3133038

[B67] Naseem H. Ahsan M. Shahid M. A. Khan N. (2018). Exopolysaccharides producing rhizobacteria and their role in plant growth and drought tolerance. J. Basic. Microbiol. 58, 1009–1022. 10.1002/jobm.201800309 30183106

[B68] Neifar M. Chouchane H. Najjari A. El Hidri D. Mahjoubi M. Ghedira K.. (2019). Genome analysis provides insights into crude oil degradation and biosurfactant production by extremely halotolerant Halomonas desertis G11 isolated from Chott El-Djerid salt-lake in Tunisian desert. Genomics 111, 1802–1814. 10.1016/j.ygeno.2018.12.003 30529640

[B69] Ogata H. Goto S. Sato K. Fujibuchi W. Bono H. Kanehisa M. (1999). KEGG: kyoto encyclopedia of genes and genomes. Nucleic. Acids. Res. 27, 29–34. 10.1093/nar/27.1.29 9847135 PMC148090

[B70] Ouertani A. Chaabouni I. Mosbah A. Long J. Barakat M. Mansuelle P.. (2018). Two new secreted proteases generate a casein-derived antimicrobial peptide in *Bacillus cereus* food born isolate leading to bacterial competition in milk. Front. Microbiol. 9:1148. 10.3389/fmicb.2018.01148 29915567 PMC5994558

[B71] Passari A. K. Rajput V. Zothanpuia Priya L. P. M. Dharne M. Dastager S. Mathew O. K.. (2019b). Draft genome sequence of plant growth-promoting endophytic *Microbacterium hydrothermale* BPSAC84, isolated from the medicinal plant Mirabilis jalapa. Microbiol. Resour. Announc. 8, e00406–e00419. 10.1128/MRA.00406-19 31147433 PMC6544190

[B72] Passari A. K. Upadhyaya K. Singh G. AbdelAzeem A. M. Thankappan S. Uthandi S.. (2019a). Enhancement of disease resistance, growth potential, and photosynthesis in tomato (*Solanum lycopersicum*) by inoculation with an endophytic actinobacterium, Streptomyces thermocarboxydus strain BPSAC147. PLoS ONE 14:e0219014. 10.1371/journal.pone.0219014 31269087 PMC6608948

[B73] Pattanapipitpaisal P. Brown N. Macaskie L. (2001). Chromate reduction and 16S rRNA identification of bacteria isolated from a Cr(VI)-contaminated site. Appl. Microbiol. Biotechnol. 57, 257–261. 10.1007/s002530100758 11693930

[B74] Payne S. M. Mey A. R. Wyckoff E. E. (2016). Vibrio iron transport: evolutionary adaptation to life in multiple environments. Microbiol. Mol. Biol. Rev. 80, 69–90. 10.1128/MMBR.00046-15 26658001 PMC4711184

[B75] Penrose D. M. Glick B. R. (2003). Methods for isolating and characterizing ACC deaminase-containing plant growth-promoting rhizobacteria. Physiol. Plant. 118, 10–15. 10.1034/j.1399-3054.2003.00086.x 12702008

[B76] Pikovskaya R. I. (1948). Mobilization of phosphorus in soil in connection with the vital activity of some microbial species. Microbiology. 17, 362–370.

[B77] Pradhan S. K. Singh N. R. Rath B. P. Thatoi H. (2016). Bacterial chromate reduction: a review of important genomic, proteomic, and bioinformatic analysis. Crit. Rev. Env Sci. Tec. 46, 1659–1703. 10.1080/10643389.2016.1258912

[B78] Ramirez-Diaz M. I. Diaz-Perez C. Vargas E. Riveros-Rosas H. Campos-Garcia J. Cervantes C. (2008). Mechanisms of bacterial resistance to chromium compounds. Biometals 21, 321–332. 10.1007/s10534-007-9121-8 17934697

[B79] Reardon T. Timmer C. P. Berdegue J. A. (2004). The rapid rise of supermarkets in developing countries: induced organizational, institutional, and technological change in agrifood systems. eJADE 1, 168–183. 10.22004/ag.econ.12005

[B80] Richter M. Rosselló-Móra R. (2009). Shifting the genomic gold standard for the prokaryotic species definition. Proc. Natl. Acad. Sci. U.S.A. 106, 19126–19131. 10.1073/pnas.0906412106 19855009 PMC2776425

[B81] Richter M. Rosselló-Móra R. Oliver-Glöckner F. Peplies J. (2016). JSpeciesWS: a web server for prokaryotic species circumscription based on pairwise genome comparison. Bioinformatics 32, 929–931. 10.1093/bioinformatics/btv681 26576653 PMC5939971

[B82] Sau G. B. Chatterjee S. Mukherjee S. K. (2010). Chromate reduction by cell-free extract of *Bacillus firmus* KUCr1. Pol. J. Microbiol. 59, 185–190. 10.33073/pjm-2010-029 21033582

[B83] Schippers A. Neretin L. N. Kallmeyer J. Ferdelman T. G. Cragg B. A. Parkes R. J.. (2005). Prokaryotic cells of the deep sub-seafloor biosphere identified as living bacteria. Nature 433, 861–864. 10.1038/nature03302 15729341

[B84] Soni S. K. Singh R. Awasthi A. Kalra A. (2014). A Cr(VI)-reducing *Microbacterium* sp. strain SUCR140 enhances growth and yield of Zea mays in Cr(VI) amended soil through reduced chromium toxicity and improves colonization of arbuscular mycorrhizal fungi. Environ. Sci. Pollut. R. 21, 1971–1979. 10.1007/s11356-013-2098-7 24014225

[B85] Souii A. Guesmi A. Ouertani R. Cherif H. Chouchane H. Cherif A. (2018). Carboxymethyl cellulase production by extremotolerant bacteria in low-cost media and application in enzymatic saccharification of stevia biomass. Waste. Biomass. Valori. 11, 2111–2122. 10.1007/s12649-018-0496-2

[B86] Spaepen S. Vanderleyden J. (2011). Auxin and plant-microbe interactions. Cold. Spring. Harb. Perspect. Biol. 3:a001438. 10.1101/cshperspect.a001438 21084388 PMC3062209

[B87] Suthanthararajan R. Ravindranath E. Chits K. Umamaheswari B. Ramesh T. Rajamam S. (2004). Membrane application for recovery and reuse of water from treated tannery wastewater. Desalination 164, 151–156. 10.1016/S0011-9164(04)00174-2

[B88] Thanikaivelan P. Rao J. R. Nair B. U. Ramasami T. (2004). Progress and recent trends in biotechnological methods for leather processing. Trends. Biotechnol. 22, 181–188. 10.1016/j.tibtech.2004.02.008 15038923

[B89] Wu Y. H. Zhou P. Cheng H. Wang C. S. Wu M. Xu X. W. (2015). Draft genome sequence of *Microbacterium profundi* Shh49T, an actinobacterium isolated from deep-sea sediment of a polymetallic nodule environment. Genome. Announc. 3:e00642-15. 10.1128/genomeA.00642-15 26067975 PMC4463539

[B90] Yang J. Roy A. Zhang Y. (2013). Protein–ligand binding site recognition using complementary binding-specific substructure comparison and sequence profile alignment. Bioinformatics 29, 2588–2595. 10.1093/bioinformatics/btt447 23975762 PMC3789548

[B91] Yang J. Zhang Y. (2015). I-TASSER server: new development for protein structure and function predictions. Nucleic. Acids. Res. 43, W174–W181. 10.1093/nar/gkv342 25883148 PMC4489253

[B92] Zeng J. Gou M. Tang Y. Q. Li G.Y. Sun Z.Y. Kida K. (2016). Effective bioleaching of chromium in tannery sludge with an enriched sulfur-oxidizing bacterial community. Bioresour. Technol. 218, 859–866. 10.1016/j.biortech.2016.07.051 27434303

