# Supplementary material for: New Plant Growth-Promoting, Chromium-Detoxifying Microbacterium Species Isolated From a Tannery Wastewater: Performance and Genomic Insights
Source: Front Bioeng Biotechnol. 2020 Jul 3;8:521. doi: 10.3389/fbioe.2020.00521 (PMC7350417; doi:10.3389/fbioe.2020.00521)
Supplement: Supplementary file 1 [file Data_Sheet_1.docx]

**New Plant Growth-Promoting, Chromium-Detoxifying *Microbacterium* Species Isolated From a Tannery Wastewater: Performance and Genomic Insights**

**Supplementary Figures :**

Content

**Figure S1.** *In vitro* plant growth-promoting traits of the TL13 strain : (a) Atmospheric nitrogen fixation ; (b) Siderophore production ; (c) Phosphate solubilization ; (d) EPS production ; (e) Cellulase activity and (f) IAA production.

**Figure S2.** Antibiogram illustrated TL13 strain sensitivity against 13 antibiotics (from left to right) : Cefoxitin (FOX : 30µg) ; Pristinamycin (PTN : 15µg) ; Neomycin (NE : 30µg) ; Rifampicin (RD : 30µg) ; Norfloxacin (NOR : 5µg) ; Streptomycin (S : 10µg) ; Erythromycin (E : 15µg) ; Cefotaxime (CTX : 30µg) ; Tetracycline (TET : 30µg) ; Vancomycin (VAN : 30µg) ; Ceftazidime (CAZ : 30µg) ; Spiramycin (SP : 100µg) and Oxytetracyclin (OT : 30µg).

**Figure S3.** Graphical representation of a taxonomic tree made with strains from the DSMZ cultivable collection according to the Gold database. A: Overview. B: Zoom on the area framed in circle in image A. Each point represents a type of strain. In red are represented the strains for which a sequencing project is in progress, the others are represented in green. The empty points represent higher taxonomic levels. The length of the branches is not significant. Figure extracted from <https://microbial-earth.namesforlife.com/>.


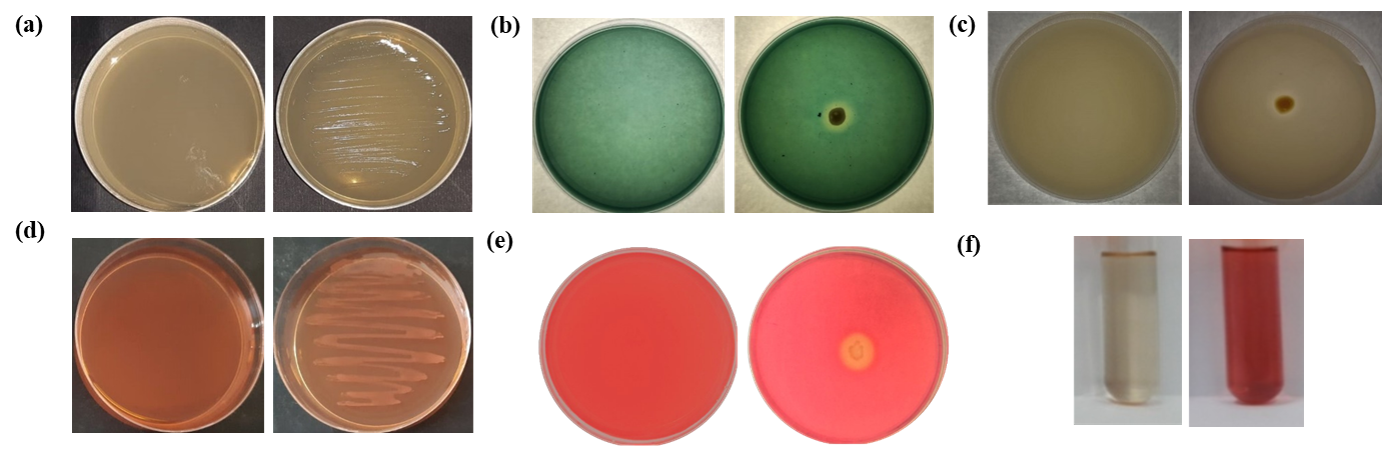


**Figure S1.** *In vitro* plant growth-promoting traits of the TL13 strain : (a) Atmospheric nitrogen fixation ; (b) Siderophore production ; (c) Phosphate solubilization ; (d) EPS production ; (e) Cellulase activity and (f) IAA production.


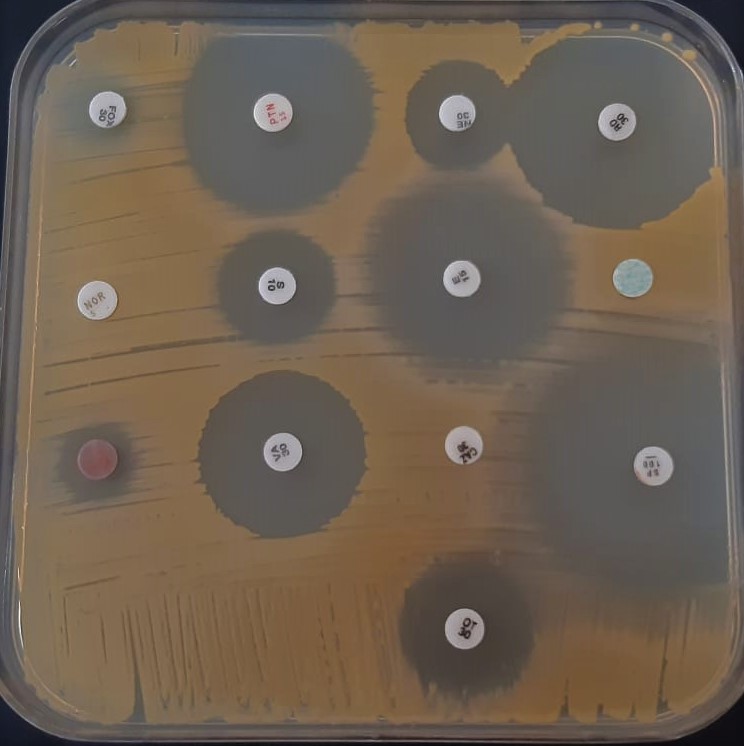


**Figure S2.** Antibiogram illustrated TL13 strain sensitivity against 13 antibiotics (from left to right) : Cefoxitin (FOX : 30µg) ; Pristinamycin (PTN : 15µg) ; Neomycin (NE : 30µg) ; Rifampicin (RD : 30µg) ; Norfloxacin (NOR : 5µg) ; Streptomycin (S : 10µg) ; Erythromycin (E : 15µg) ; Cefotaxime (CTX : 30µg) ; Tetracycline (TET : 30µg) ; Vancomycin (VAN : 30µg) ; Ceftazidime (CAZ : 30µg) ; Spiramycin (SP : 100µg) and Oxytetracyclin (OT : 30µg).


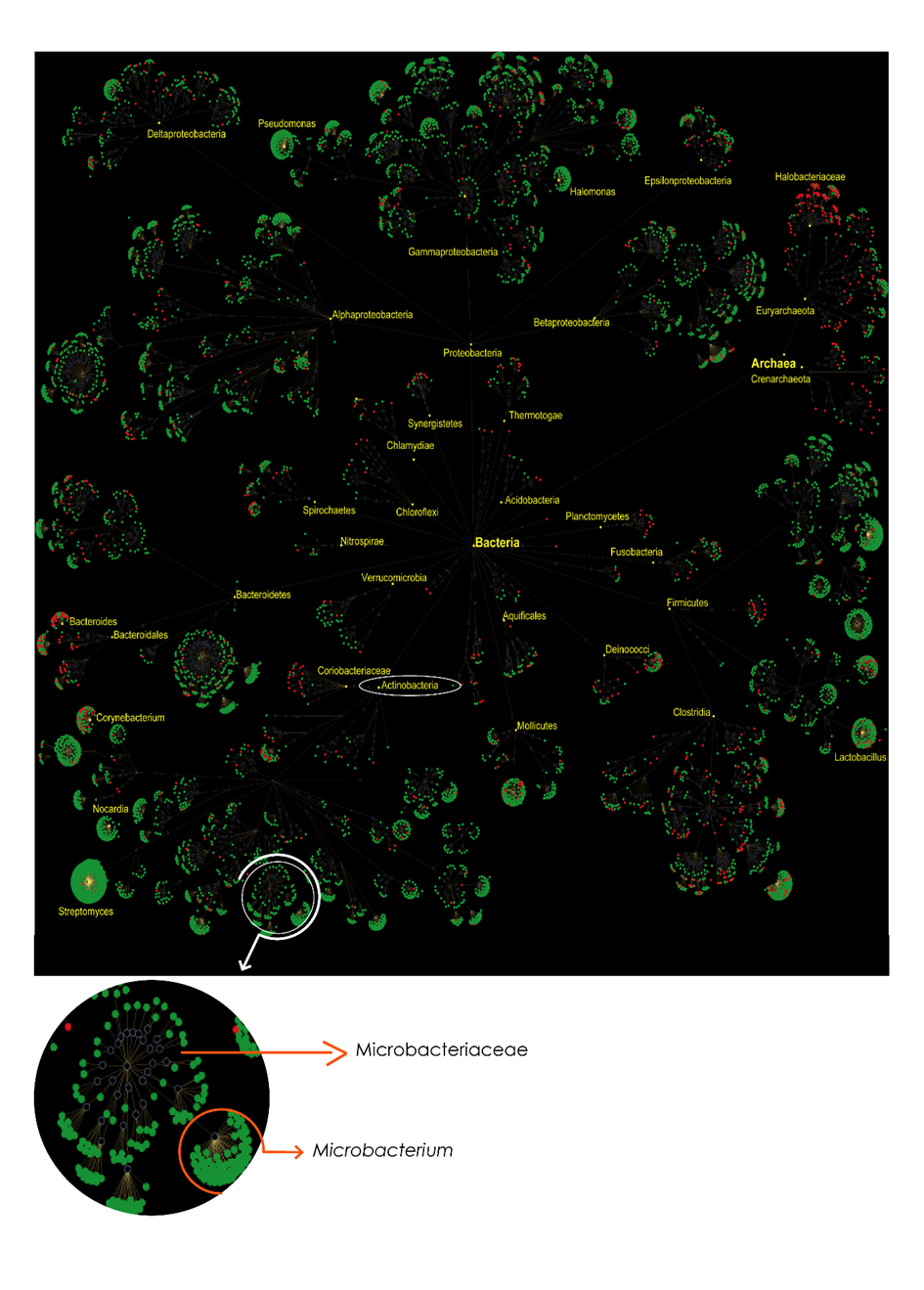
**Figure S3.** Graphical representation of a taxonomic tree made with strains from the DSMZ cultivable collection according to the Gold database. A: Overview. B: Zoom on the area framed in circle in image A. Each point represents a type of strain. In red are represented the strains for which a sequencing project is in progress, the others are represented in green. The empty points represent higher taxonomic levels. The length of the branches is not significant. Figure extracted from <https://microbial-earth.namesforlife.com/>.

**Supplementary Tables :**

Content

**Table S1*.*** Result of antibiotic sensitivity test.

**Table S2.** The RAST Iron acquisition metabolic reconstruction of the submitted genome of *Microbacterium metallidurans* TL13 (A) compared to *Cupriavidus metallidurans* CH34 (B).

**Table S3.** KEGG map biosynthesis of siderophore group nonribosomal peptides for *Microbacterium metallidurans* TL13 and *Cupriavidus metallidurans* CH34

**Table S1*.*** Result of antibiotic sensitivity test.

| Antibiotic | Code | Disk content (µg) | Antibiotic group | Resistance or Sensitivity | Inhibition zone (mm) |
| --- | --- | --- | --- | --- | --- |
| Cefoxitin | FOX | 30 | Cephamycin | Sensitive | 14 |
| Pristinamycin | PTN | 15 | Streptogramin | Sensitive | 33 |
| Neomycin | NE | 30 | Aminoglycoside | Sensitive | 20 |
| Rifampicin | RD | 30 | Rifampicin | Sensitive | 37 |
| Norfloxacin | NOR | 5 | Fluoroquinolone | Resistant | - |
| Streptomycin | S | 10 | Aminoglycoside | Sensitive | 21 |
| Erythromycin | E | 15 | Macrolide | Sensitive | 35 |
| Cefotaxime | CTX | 30 | β-lactam | Resistant | - |
| Tetracycline | TET | 30 | Tetracycline | Sensitive | 19 |
| Vancomycin | VAN | 30 | Glycopeptide | Sensitive | 28 |
| Ceftazidime | CAZ | 30 | β-lactam | Resistant | - |
| Spiramycin | SP | 100 | Macrolide | Sensitive | 42 |
| Oxytetracyclin | OT | 30 | Oxytetracyclin | Sensitive | 22 |

**Table S2.** The RAST Iron acquisition metabolic reconstruction of the submitted genome of *Microbacterium metallidurans* TL13 (A) compared to *Cupriavidus metallidurans* CH34 (B).

| **Presence** | **Category** | **Subcategory** | **Subsystem** | **Role** | **Organism A** | **SS active A** | **SS active B** |
| --- | --- | --- | --- | --- | --- | --- | --- |
| A | Iron acquisition and metabolism | no subcategory | Ferrous iron transporter EfeUOB, low-pH-induced | Ferrous iron transport periplasmic protein EfeO, contains peptidase-M75 domain and (frequently) cupredoxin-like domain | [fig\|69370.7.peg.2712](http://rast.theseed.org/FIG/seedviewer.cgi?page=Annotation&feature=fig\|69370.7.peg.2712) | yes | no |
| A | Iron acquisition and metabolism | no subcategory | Ferrous iron transporter EfeUOB, low-pH-induced | Ferrous iron transport permease EfeU | [fig\|69370.7.peg.2713](http://rast.theseed.org/FIG/seedviewer.cgi?page=Annotation&feature=fig\|69370.7.peg.2713) | yes | no |
| A | Iron acquisition and metabolism | no subcategory | Ferrous iron transporter EfeUOB, low-pH-induced | Ferrous iron transport peroxidase EfeB | [fig\|69370.7.peg.2711](http://rast.theseed.org/FIG/seedviewer.cgi?page=Annotation&feature=fig\|69370.7.peg.2711) | yes | no |
| A | Iron acquisition and metabolism | no subcategory | Heme, hemin uptake and utilization systems in GramPositives | Heme ABC transporter, ATPase component HmuV | [fig\|69370.7.peg.3272](http://rast.theseed.org/FIG/seedviewer.cgi?page=Annotation&feature=fig\|69370.7.peg.3272) | yes | no |
| A | Iron acquisition and metabolism | no subcategory | Heme, hemin uptake and utilization systems in GramPositives | Heme ABC transporter, cell surface heme and hemoprotein receptor HmuT | [fig\|69370.7.peg.3270](http://rast.theseed.org/FIG/seedviewer.cgi?page=Annotation&feature=fig\|69370.7.peg.3270) | yes | no |
| A | Iron acquisition and metabolism | no subcategory | Heme, hemin uptake and utilization systems in GramPositives | Heme ABC transporter, permease protein HmuU | [fig\|69370.7.peg.3271](http://rast.theseed.org/FIG/seedviewer.cgi?page=Annotation&feature=fig\|69370.7.peg.3271) | yes | no |
| A | Iron acquisition and metabolism | no subcategory | Heme, hemin uptake and utilization systems in GramPositives | Heme oxygenase (EC 1.14.99.3) | [fig\|69370.7.peg.1850](http://rast.theseed.org/FIG/seedviewer.cgi?page=Annotation&feature=fig\|69370.7.peg.1850), [fig\|69370.7.peg.3267](http://rast.theseed.org/FIG/seedviewer.cgi?page=Annotation&feature=fig\|69370.7.peg.3267) | yes | no |
| A | Iron acquisition and metabolism | no subcategory | Heme, hemin uptake and utilization systems in GramPositives | Hemoglobin, heme-dependent two component system sensory histidine kinase ChrS | [fig\|69370.7.peg.1952](http://rast.theseed.org/FIG/seedviewer.cgi?page=Annotation&feature=fig\|69370.7.peg.1952) | yes | no |
| A | Iron acquisition and metabolism | no subcategory | Heme, hemin uptake and utilization systems in GramPositives | Hemoglobin-dependent two component system response regulator HrrA | [fig\|69370.7.peg.1008](http://rast.theseed.org/FIG/seedviewer.cgi?page=Annotation&feature=fig\|69370.7.peg.1008) | yes | no |
| A | Iron acquisition and metabolism | no subcategory | Heme, hemin uptake and utilization systems in GramPositives | Hemoglobin-dependent two component system, sensory histidine kinase HrrS | [fig\|69370.7.peg.1009](http://rast.theseed.org/FIG/seedviewer.cgi?page=Annotation&feature=fig\|69370.7.peg.1009) | yes | no |
| A | Iron acquisition and metabolism | no subcategory | Heme, hemin uptake and utilization systems in GramPositives | Iron compound ABC uptake transporter substrate-binding protein PiaA | [fig\|69370.7.peg.2515](http://rast.theseed.org/FIG/seedviewer.cgi?page=Annotation&feature=fig\|69370.7.peg.2515) | yes | no |
| A | Iron acquisition and metabolism | no subcategory | Heme, hemin uptake and utilization systems in GramPositives | Iron-dependent repressor IdeR/DtxR | [fig\|69370.7.peg.755](http://rast.theseed.org/FIG/seedviewer.cgi?page=Annotation&feature=fig\|69370.7.peg.755) | yes | no |

**Table S2.** The RAST Iron acquisition metabolic reconstruction of the submitted genome of *Microbacterium metallidurans* TL13 (A) compared to *Cupriavidus metallidurans* CH34 (B) (continued).

| **Presence** | **Category** | **Subcategory** | **Subsystem** | **Role** | **Organism A** | **SS active B** | **SS active A** |
| --- | --- | --- | --- | --- | --- | --- | --- |
| B | Iron acquisition and metabolism | Siderophores | Iron siderophore sensor & receptor system | FIG006045: Sigma factor, ECF subfamily | [fig\|266264.4.peg.1525](http://rast.theseed.org/FIG/seedviewer.cgi?page=Annotation&feature=fig\|266264.4.peg.1525), [fig\|266264.4.peg.4388](http://rast.theseed.org/FIG/seedviewer.cgi?page=Annotation&feature=fig\|266264.4.peg.4388) | yes | no |
| B | Iron acquisition and metabolism | Siderophores | Iron siderophore sensor & receptor system | Iron siderophore receptor protein | [fig\|266264.4.peg.4386](http://rast.theseed.org/FIG/seedviewer.cgi?page=Annotation&feature=fig\|266264.4.peg.4386) | yes | no |
| B | Iron acquisition and metabolism | Siderophores | Iron siderophore sensor & receptor system | Iron siderophore sensor protein | [fig\|266264.4.peg.1524](http://rast.theseed.org/FIG/seedviewer.cgi?page=Annotation&feature=fig\|266264.4.peg.1524), [fig\|266264.4.peg.4387](http://rast.theseed.org/FIG/seedviewer.cgi?page=Annotation&feature=fig\|266264.4.peg.4387) | yes | no |
| B | Iron acquisition and metabolism | Siderophores | Siderophore assembly kit | 2,3-dihydroxybenzoate-AMP ligase (EC 2.7.7.58) | [fig\|266264.4.peg.4166](http://rast.theseed.org/FIG/seedviewer.cgi?page=Annotation&feature=fig\|266264.4.peg.4166) | yes | no |
| B | Iron acquisition and metabolism | Siderophores | Siderophore assembly kit | TonB-dependent hemin receptor | [fig\|266264.4.peg.5751](http://rast.theseed.org/FIG/seedviewer.cgi?page=Annotation&feature=fig\|266264.4.peg.5751) | yes | no |
| B | Iron acquisition and metabolism | no subcategory | Encapsulating protein for DyP-type peroxidase and ferritin-like protein oligomers | [NiFe] hydrogenase nickel incorporation protein HypA | [fig\|266264.4.peg.1690](http://rast.theseed.org/FIG/seedviewer.cgi?page=Annotation&feature=fig\|266264.4.peg.1690), [fig\|266264.4.peg.1937](http://rast.theseed.org/FIG/seedviewer.cgi?page=Annotation&feature=fig\|266264.4.peg.1937) | yes | no |
| B | Iron acquisition and metabolism | no subcategory | Encapsulating protein for DyP-type peroxidase and ferritin-like protein oligomers | [NiFe] hydrogenase nickel incorporation-associated protein HypB | [fig\|266264.4.peg.1689](http://rast.theseed.org/FIG/seedviewer.cgi?page=Annotation&feature=fig\|266264.4.peg.1689), [fig\|266264.4.peg.1938](http://rast.theseed.org/FIG/seedviewer.cgi?page=Annotation&feature=fig\|266264.4.peg.1938) | yes | no |
| B | Iron acquisition and metabolism | no subcategory | Heme, hemin uptake and utilization systems in GramNegatives | ABC-type Fe3+-siderophore transport system permease component | [fig\|266264.4.peg.3180](http://rast.theseed.org/FIG/seedviewer.cgi?page=Annotation&feature=fig\|266264.4.peg.3180) | yes | no |
| B | Iron acquisition and metabolism | no subcategory | Heme, hemin uptake and utilization systems in GramNegatives | ABC-type hemin transport system, ATPase component | [fig\|266264.4.peg.5756](http://rast.theseed.org/FIG/seedviewer.cgi?page=Annotation&feature=fig\|266264.4.peg.5756) | yes | no |
| B | Iron acquisition and metabolism | no subcategory | Heme, hemin uptake and utilization systems in GramNegatives | Ferric siderophore transport system, biopolymer transport protein ExbB | [fig\|266264.4.peg.942](http://rast.theseed.org/FIG/seedviewer.cgi?page=Annotation&feature=fig\|266264.4.peg.942) | yes | no |
| B | Iron acquisition and metabolism | no subcategory | Heme, hemin uptake and utilization systems in GramNegatives | Ferric siderophore transport system, periplasmic binding protein TonB | [fig\|266264.4.peg.2675](http://rast.theseed.org/FIG/seedviewer.cgi?page=Annotation&feature=fig\|266264.4.peg.2675) | yes | no |
| B | Iron acquisition and metabolism | no subcategory | Heme, hemin uptake and utilization systems in GramNegatives | Hemin ABC transporter, permease protein | [fig\|266264.4.peg.5755](http://rast.theseed.org/FIG/seedviewer.cgi?page=Annotation&feature=fig\|266264.4.peg.5755) | yes | no |
| B | Iron acquisition and metabolism | no subcategory | Heme, hemin uptake and utilization systems in GramNegatives | Hemin transport protein HmuS | [fig\|266264.4.peg.5753](http://rast.theseed.org/FIG/seedviewer.cgi?page=Annotation&feature=fig\|266264.4.peg.5753) | yes | no |
| B | Iron acquisition and metabolism | no subcategory | Heme, hemin uptake and utilization systems in GramNegatives | Hemin uptake protein | [fig\|266264.4.peg.2678](http://rast.theseed.org/FIG/seedviewer.cgi?page=Annotation&feature=fig\|266264.4.peg.2678) | yes | no |
| B | Iron acquisition and metabolism | no subcategory | Heme, hemin uptake and utilization systems in GramNegatives | Paraquat-inducible protein A | [fig\|266264.4.peg.4475](http://rast.theseed.org/FIG/seedviewer.cgi?page=Annotation&feature=fig\|266264.4.peg.4475), [fig\|26626](http://rast.theseed.org/FIG/seedviewer.cgi?page=Annotation&feature=fig\|266264.4.peg.986) | yes | no |

**Table S3.** KEGG map biosynthesis of siderophore group nonribosomal peptides for *Microbacterium metallidurans* TL13 and *Cupriavidus metallidurans* CH34.

| **KEGG map** | ***Microbacterium trichothecenolyticum* TL13** | ***Cupriavidus metallidurans*** CH34 |
| --- | --- | --- |
| Biosynthesis of siderophore group nonribosomal peptides | 3 (42.9 %) | [4](http://rast.theseed.org/FIG/seedviewer.cgi?page=Kegg&organism=266264.4&comparison_organism=69370.7&comparison_organism=69370.7&map=01053) (57.1 %) |
| Phenylalanine, tyrosine and tryptophan biosynthesis | 18 (58.1 %) | [21](http://rast.theseed.org/FIG/seedviewer.cgi?page=Kegg&organism=266264.4&comparison_organism=69370.7&comparison_organism=69370.7&map=00400) (67.7 %) |
